# Value of c-MET and Associated Signaling Elements for Predicting Outcomes and Targeted Therapy in Penile Cancer

**DOI:** 10.3390/cancers14071683

**Published:** 2022-03-25

**Authors:** Anita Thomas, Kimberly Sue Slade, Roman A. Blaheta, Sascha D. Markowitsch, Philipp Stenzel, Katrin E. Tagscherer, Wilfried Roth, Mario Schindeldecker, Martin Michaelis, Florian Rothweiler, Jaroslav Cinatl, Robert Dotzauer, Olesya Vakhrusheva, Maarten Albersen, Axel Haferkamp, Eva Juengel, Jindrich Cinatl, Igor Tsaur

**Affiliations:** 1 Department of Urology and Pediatric Urology, University Medicine Mainz, 55131 Mainz, Germany; kimberlysue.slade@unimedizin-mainz.de (K.S.S.); roman.blaheta@unimedizin-mainz.de (R.A.B.); sascha.markowitsch@unimedizin-mainz.de (S.D.M.); robert.dotzauer@unimedizin-mainz.de (R.D.); olesya.vakhrusheva@unimedizin-mainz.de (O.V.); axel.haferkamp@unimedizin-mainz.de (A.H.); eva.juengel@unimedizin-mainz.de (E.J.); igor.tsaur@unimedizin-mainz.de (I.T.); 2 Department of Pathology, University Medicine Mainz, 55131 Mainz, Germany; philipp.stenzel@unimedizin-mainz.de (P.S.); katrin.tagscherer@unimedizin-mainz.de (K.E.T.); wilfried.roth@unimedizin-mainz.de (W.R.); mario.schindeldecker@unimedizin-mainz.de (M.S.); 3 Industrial Biotechnology Centre, School of Biosciences, University of Kent, Canterbury CT2 7NJ, UK; m.michaelis@kent.ac.uk; 4 Institute of Medical Virology, Goethe-University, 60596 Frankfurt am Main, Germany; f.rothweiler@kinderkrebsstiftung-frankfurt.de (F.R.); ja.cinatl@kinderkrebsstiftung-frankfurt.de (J.C.); cinatl@em.uni-frankfurt.de (J.C.J.); 5 Dr. Petra Joh-Forschungshaus, 60528 Frankfurt am Main, Germany; 6 Department of Urology, University Hospitals Leuven, 28046 Leuven, Belgium; maarten.albersen@uzleuven.be

**Keywords:** penile cancer, squamous cell carcinoma, c-MET, resistant cell lines, marker, targeted therapy, tivantinib, cabozantinib

## Abstract

**Simple Summary:**

No relevant improvement in patient outcomes could be achieved in the last decade in metastasized penile cancer due to insufficient identification of molecular hubs crucial for tumor evolution. We investigated the potential of the cellular receptor c-MET and selected other proteins linked to its activity to predict outcomes and for exploitation in targeted treatment. Assessing tumor tissue as well as primary cells both naïve and resistant to systemic drugs, we illustrate the most promising role of c-MET. Indeed, its elevated expression was strongly associated with inferior tumor-related survival. Moreover, its upregulation in treatment-resistant cell lines compared to naïve cells was observed. Treating cell lines with the c-MET inhibitors cabozantinib and tivantinib mediated an effective decrease in cell growth, while the first agent was more efficacious in the naïve cells and the second agent in the resistant cells. Therefore, c-MET blockade warrants further investigation in the setting of metastasized penile cancer.

**Abstract:**

Whereas the lack of biomarkers in penile cancer (PeCa) impedes the development of efficacious treatment protocols, preliminary evidence suggests that c-MET and associated signaling elements may be dysregulated in this disorder. In the following study, we investigated whether c-MET and associated key molecular elements may have prognostic and therapeutic utility in PeCa. Formalin-fixed, paraffin-embedded tumor tissue from therapy-naïve patients with invasive PeCa was used for tissue microarray (TMA) analysis. Immunohistochemical staining was performed to determine the expression of the proteins c-MET, PPARg, β-catenin, snail, survivin, and n-MYC. In total, 94 PeCa patients with available tumor tissue were included. The median age was 64.9 years. High-grade tumors were present in 23.4%, and high-risk HPV was detected in 25.5%. The median follow-up was 32.5 months. High expression of snail was associated with HPV-positive tumors. Expression of β-catenin was inversely associated with grading. In both univariate COX regression analysis and the log-rank test, an increased expression of PPARg and c-MET was predictive of inferior disease-specific survival (DSS). Moreover, in multivariate analysis, a higher expression of c-MET was independently associated with worse DSS. Blocking c-MET with cabozantinib and tivantinib induced a significant decrease in viability in the primary PeCa cell line UKF-PeC3 isolated from the tumor tissue as well as in cisplatin- and osimertinib-resistant sublines. Strikingly, a higher sensitivity to tivantinib could be detected in the latter, pointing to the promising option of utilizing this agent in the second-line treatment setting.

## 1. Introduction

Cancer treatment based on the principle of interfering with molecules crucial for tumor cell growth, proliferation, and dissemination capacity has achieved a stunning improvement in clinical outcomes in a plethora of malignancies lately. For instance, targeted therapies have strikingly expanded the treatment armamentarium of advanced thyroid, breast, colorectal, lung, and ovarian cancers as well as leukemia [1,2]. Among genitourinary cancers, the clinical benefit of targeting vascular endothelial (VEGF) and platelet-derived growth factor (PDGF) receptors by tyrosine kinase inhibitors is well established in renal cell carcinoma [3]. In patients with metastatic bladder cancer progressing after platinum-based chemotherapy and immune checkpoint inhibitors, targeted approaches with the antibody–drug conjugates enfortumab vedotin and acituzumab govitecan directed against Nectin-4 and Trop-2 have most recently shown promising objective response rates (ORRs) of 44% and 27%, respectively [4,5]. By mischance, no breakthrough in the efficacy of systemic protocols has been observed yet for another malignant disorder traditionally treated by cytotoxic therapeutics—penile cancer (PeCa) [6].

Several obstacles hamper the development of efficacious concepts of management in advanced PeCa. With an incidence of 0.45–1.7 per 100.000 population in European countries and the USA [7], it represents an orphan disease mainly deprived of financial support by pharmaceutical companies, thus failing appropriate assessment in clinical trials. In addition, small sample-sized databases and biobanks as well as the scarce availability of cell lines and xenograft models impede profound translational research which might yield the groundwork for testing novel treatment concepts. Therefore, males with disseminated PeCa are still being offered cisplatin-based protocols, achieving an ORR of 50% in the neoadjuvant setting at best [8], whereas no efficacious therapy exists upon progression. Thus, the medical need for identifying indispensable elements of the tumor molecular machinery which might be pharmacologically targeted is currently unmet more than ever.

In this context, the scientific spotlight is increasingly shone on the receptor tyrosine kinase cellular mesenchymal-epithelial transcription factor (c-MET) and the associated signaling apparatus. Contemporary evidence points to sustained c-MET stimulation, overexpression, or mutation in a variety of malignancies [9]. Since this pathway represents one of the critical hubs steering tumor formation, progression, and dissemination, its targeting represents an attractive option for systemic management of advanced cancers [10]. Previously, we illuminated the role of the PI3K/mTOR/AKT signaling cascade [11], which is one of the downstream pathways modulated by c-MET. The current assessment sheds light on the potential of c-MET itself and that of its signaling interacting molecules β-catenin, snail, PPAR-γ, n-myc, and survivin, for which engagement in neoplastic processes has been reported [12,13,14,15,16], to be exploited as biomarkers and novel therapeutic targets in advanced PeCa.

## 2. Materials and Methods

### 2.1. Study Cohort

After obtaining ethical board approval, we included PeCa patients with available formalin-fixed, paraffin-embedded tissue samples provided by the tissue bank of the University Medical Center Mainz in accordance with the regulations of the tissue bank. All patients with non-metastatic invasive penile cancer treated from 1980 until 2021 were included. Patient data including age at diagnosis, concomitant diseases, primary tumor surgery, tumor grading, pathologic T (pT) and N (pN) stage, HPV status, disease recurrence, receipt of adjuvant therapy, and survival data were collected. Pathological staging was documented according to the currently valid World Health Organization (WHO) and TNM classifications. Four older cases with a missing T-stage classification were included because the pathology report described invasive cancer extension to or beyond the subepithelial connective tissue.

### 2.2. Tissue Microarray Construction and Immunohistochemical Staining

A tissue microarray (TMA) for 94 patients with invasive PeCa was constructed as previously described [11]. The presence of the high-risk (hr)HPV DNA in tumor tissue and cell lines was analyzed by PCT-based Sanger sequencing, as previously described [11]. For immunohistochemical staining, the following antibodies were applied: Peroxisome proliferator-activated receptor gamma (PPARG) (C26H12), Cell Signaling; Survivin (71G4B7), Cell Signaling; n-myc (polyclonal), Abcam; Snail (C15D3), Cell Signaling; c-MET (D1C2), Cell Signaling; β-catenin (β-Catenin-1), Agilent/Dako.

Biomarker expression was determined by the product of staining intensity and percentage of positively stained cells, ranging from 0 to 9 [17]. The selection of cut-off scores was based on receiver operating characteristic (ROC) curve analysis (Supplemental Table S1) [18].

### 2.3. Cell Culture and Induction of Drug Resistance

The UKF-PeC3 cell line was generated from a patient with a histopathologically and immunohistochemically confirmed pT3 pN0 L0 G2 R0, HPV DNA Typ 33 (high risk) positive PeCa, as previously described [11]. The cell line was tested to be negative for hrHPV DNA after establishment. Drug-adapted cell lines were derived from the Resistant Cancer Cell Line (RCCL) collection [19]. UKF-PeC3 cells were adapted to growth in the presence of 2 μg/mL cisplatin (UKF-PeC3^r^CIS^2^) or 2 μM osimertinib (UKF-PeC3^r^OSI^2^) by continuous exposure to increasing concentrations of the respective drugs, as described before [20]. The half-maximal inhibitory concentration (IC50) of cisplatin or osimertinib in tumor cells was investigated to verify drug resistance.

UKF-PeC3 and resistant cell lines UKF-PeC3^r^CIS^2^ and UKF-PeC3^r^OSI^2^ were grown at 37 °C in Iscove’s modified Dulbecco’s medium (IMDM) supplemented with 10% fetal calf serum (FCS, Gibco, Karlsruhe, Germany), 1% Anti/Anti (Gibco, Darmstadt, Germany), and 1% glutamax (Gibco, Darmstadt, Germany), containing 2 μg/mL cisplatin (UKF-PeC3^r^CIS^2^) or 2 μM osimertinib (UKF-PeC3^r^OSI^2^) in respective resistant cell lines. Cell line authentication and validation were performed by short tandem repeat (STR) profiling (Institute of Legal Medicine, Frankfurt, Germany). Exome sequencing did not reveal mutations in the c-MET gene (GeneXPro GmbH, Frankfurt, Germany).

### 2.4. Drugs

Cisplatin was purchased from Selleckchem (via Absource Diagnostics GmbH, Munich, Germany) and osimertinib from MedChemExpress (via Hycultec, Beutelsbach, Germany). Cabozantinib and tinvantinib, both obtained from Selleckchem (via Absource Diagnostics GmbH, Munich, Germany), were used for pharmacological inhibition. Cabozantinib, a receptor tyrosine kinase inhibitor (TKI) that targets multiple tyrosine kinases including VEGFR2, RET, AXL, FLT3, c-KIT, and c-MET, is clinically approved for the treatment of medullary thyroid cancer, renal cell carcinoma, and hepatocellular carcinoma [21]. Tivantinib, a selective inhibitor of unactivated c-MET and its self-phosphorylation, has been successful in phase II trials; however, it failed in two phase III trials for second-line treatment of advanced high-Met hepatocellular carcinoma [22]. Drugs were used at non-toxic concentrations evaluated by trypan blue staining.

### 2.5. Tumor Cell Growth

Cell growth was assessed by using the 3-(4,5-dimethylthiazol-2-yl)-2,5-diphenyltetrazolium bromide (MTT) dye reduction assay. Tumor cells were seeded into 96-well microtiter plates containing serial dilutions of the indicated substances. After 24, 48, and 72 h incubation, MTT (0.5 mg/mL) was added for 4 h. Cells were lysed in a solubilization buffer containing 10% SDS in 0.01 M HCl. The plates were then incubated overnight at 37 °C. Absorbance for each well was detected at 570 nm using a microplate enzyme-linked immunosorbent assay (ELISA) reader. In order to illustrate the kinetics of dose–response, 24 h data were set to 100%.

### 2.6. SDS-PAGE/Western Blot

Protein concentration was determined by the bicinchoninic acid (BCA) assay. Tumor cell lysates were applied to a polyacrylamide gel and were separated for 10 min at 80 V and for 1 h at 120 V. Proteins were transferred to a nitrocellulose membrane which was blocked with non-fat dry milk for 60 min. The membranes were then incubated overnight with the following primary antibodies directed against: snail (clone C15D3), rabbit immunoglobulin IgG1, dilution 1:1000, Cell Signaling; PPARg (C26H12), rabbit immunoglobulin IgG1, dilution 1:1000, Cell Signaling; survivin (clone 71G4B7), rabbit immunoglobulin IgG1, dilution 1:1000; n-myc (clone 51705S), rabbit immunoglobulin IgG1, dilution 1:1000; β-catenin (clone D10A8), rabbit immunoglobulin IgG1, dilution 1:1000; c-MET (clone D1C2), rabbit immunoglobulin IgG1, dilution 1:1000. Horseradish peroxidase-conjugated goat-anti-rabbit IgG (dilution 1:1000, Dako, Glosturp, Denmark) served as the secondary antibody.

Antibody complexes were detected using the ECL reagent (AC2204, Azure Biosystems, Munich, Germany) and subsequently visualized by Sapphire Imager (Azure Biosystems, Munich, Germany). Membranes were stained by Coomassie brilliant blue R-250 and measured by Sapphire Imager for an internal loading control. The protein intensity/whole protein intensity ratio was determined and expressed in percentage, related to untreated controls, set to 100%.

### 2.7. Statistics

All statistical analyses were performed within the R environment for statistical computing (version 4.0.3, R Foundation for Statistical Computing, Vienna, Austria). The non-parametric Mann–Whitney U test was used to compare differences between two independent groups when dependent variables were either ordinal or continuous. Categorical variables were compared using Fisher’s exact test. Overall survival (OS) was defined as the interval between surgical resection and death, regardless of etiology or the last follow-up. Recurrence-free survival (RFS) was defined as the interval from surgical resection to the detection of progression or death from any cause. Disease-specific survival (DSS) was calculated as the interval from surgical resection to death caused by disease. Last follow-up and death of other causes were considered censored events. Association between patient survival and biomarker expression was calculated by the log-rank test and Kaplan–Meier plots. Univariate and multivariate Cox regression models were used for statistical analysis by calculation of the hazard ratios and 95% confidence intervals. Biomarker correlations were calculated using Spearman’s coefficient analysis.

In vitro experiments were performed at least three times. Statistical significance was calculated with the GraphPad Prism 7.0 (GraphPad Software Inc., San Diego, CA, USA) two-way ANOVA test and two-sided t-test. Differences with error probabilities of *p* < 0.05 were considered significant.

## 3. Results

### 3.1. Clinicopathological Features of Patients

We identified a total of 94 PeCa patients with available tissue of invasive primary PeCa treated from 1980 until 2021 at our institution (Table 1). The median age at diagnosis was 67 years (interquartile range, 31–90 years). As primary surgery, 8 patients (8.5%) had circumcision, 5 patients (5.3%) had wide local tumor excision, 55 patients (58.5%) had partial penectomy, and 25 patients (26.6%) underwent total penectomy. A total of 22 patients (23.4%) presented with high-grade tumors, and 17 patients (18.1%) had positive lymph nodes (pN1–pN3). High-risk HPV was identified in 24 cases (25.5%).

### 3.2. Expression of Protein Candidates in the Tumor Tissue

β-Catenin expression was elevated in 41 patients (43.6%; Supplemental Table S2). Its expression was inversely associated with tumor grading (*p* ≤ 0.05) (Supplemental Figure S1), whereas it was independent of pT/N stage, age at diagnosis, comorbidities, or HPV detection.

Survivin expression was increased in 23 males (24.5%; Supplemental Table S2). Moreover, higher survivin expression values were more common in patients with HPV infection and obesity (both *p* ≤ 0.05; Supplemental Figure S1).

**Figure 1:**
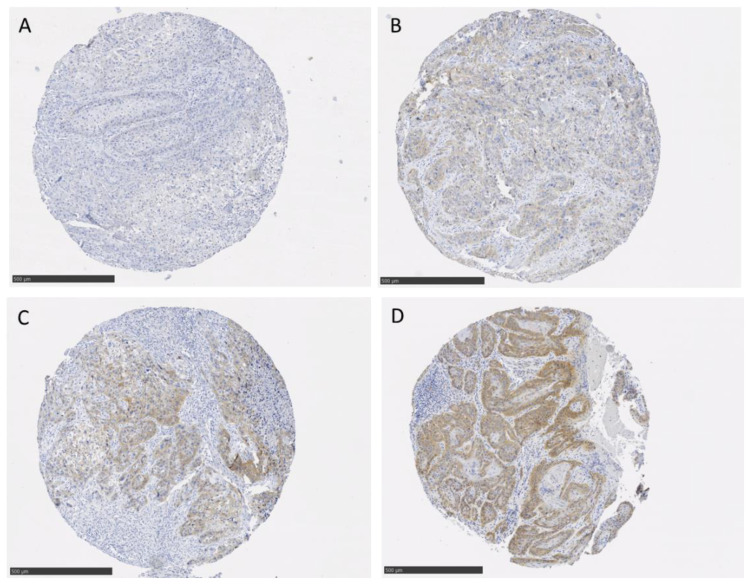
Representative immunohistochemical images of PeCa TMA tissue stained with c-MET antibody including different intensity scores of 0 (**A**), 1 (**B**), 2 (**C**), and 3 (**D**); scale bar = 100 µm.

Furthermore, c-MET expression was augmented in 41 men (43.6%; Supplemental Table S2, representative images of c-MET in Figure 1).

In univariate analysis, there were no significant associations between the protein expression level of the biomarker candidates c-MET, n-myc, PPARg, and snail and clinicopathological features. In contrast to β-catenin, there was no association between c-MET or PPARg expression and HPV infection (Supplemental Figure S2).

### 3.3. Biomarker Correlations

c-MET expression was positively associated with PPARg and survivin expression (*p* = 0.03 and *p* = 0.01, respectively). Further, a positive association between snail and β-catenin was detected (*p* = 0.01) (Supplemental Figure S3).

### 3.4. Follow-Up

The median follow-up of the current study cohort was 32.5 months. Overall, 28 patients (29.8%) developed recurrent disease, of which 18 patients (19.2%) had local recurrence and 10 patients (10.2%) had systemic recurrence (inguinal and/or visceral metastases). A total of 16 patients (17.0%) underwent adjuvant chemotherapy, 1 patient (1.1%) received radiotherapy, and 2 patients (2.6%) were treated with chemoradiotherapy. Overall, 30 patients (31.8%) died during follow-up. Twelve patients succumbed to PeCa (12.8%).

In Kaplan–Meier analysis regarding clinicopathological parameters, age at diagnosis (>65 years) was associated with shortened RFS (*p* = 0.02). Furthermore, node-positive disease (N1–N3) was associated with decreased DSS (*p* = 0.001) (Supplemental Figure S4). There was no association between HPV infection and survival data (Supplemental Figure S5).

**Figure 2:**
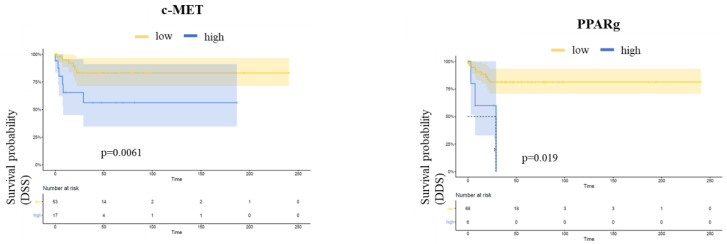
Kaplan–Meier plots of DSS according to c-MET and PPARg. Elevated biomarker expressions were associated with worse DSS (*p* = 0.019 and *p* = 0.0061, respectively).

In Kaplan–Meier analysis regarding biomarker expression, a higher expression of c-MET and PPARg was associated with worse DSS (*p* = 0.019 and *p* = 0.0061, respectively), whereas no difference in RFS or OS could be detected (Figure 2).

Accordingly, in the multivariate COX regression analysis including biomarkers associated with survival or grading as well as relevant histopathological parameters, higher c-MET expression was an independent predictor of worse DSS (HR 5.03; 95% CI, 1.08–23.32, *p* = 0.04). Furthermore, a trend for unfavorable DSS associated with a higher expression of PPARg was observed (*p* = 0.06) (Table 2).

In accordance with the univariate analysis, lymph node-positive disease (N1–N3) was an independent predictor of inferior DSS in multivariate analysis (HR 14.09; 95% CI, 3.29–60.43; *p* < 0.001) (Table 2).

### 3.5. Induction of Resistance to Cisplatin and Osimertinib

**Figure 3:**
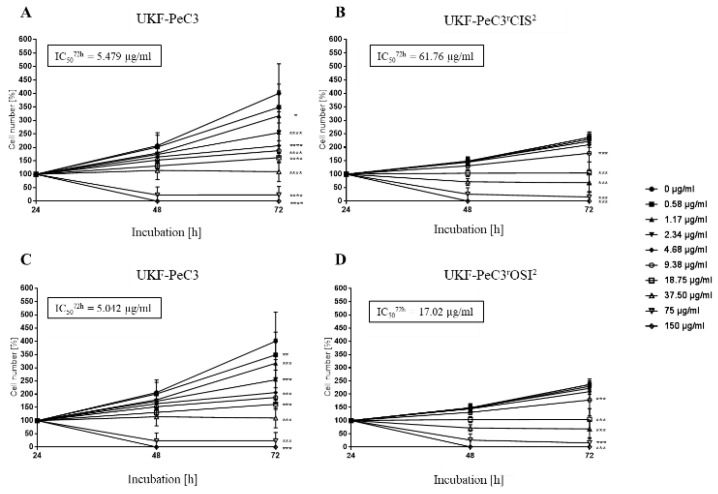
Tumor cell growth of UKF-PeC3 (**A**,**C**), UKF-PeC3^r^CIS^2^ (**B**), and PeC3^r^OSI^2^ (**D**) cells after 24, 48, and 72 h treatment with ascending cisplatin (**A**,**B**) or osimertinib (**C**,**D**) concentrations. The cell number was set to 100% after 24 h incubation. The IC_50_ of cisplatin and osimertinib after 72 h treatment is specified. Error bars indicate the standard deviation (SD). Significant difference from untreated control: * = *p* ≤ 0.05, ** = *p* ≤ 0.01, *** = *p* ≤ 0.001.

Therapy-naïve UKF-PeC3 cells were adapted to growth in the presence of 2 μg/mL cisplatin (UKF-PeC3^r^CIS^2^) or 2 μM osimertinib (UKF-PeC3^r^OSI^2^). The IC50 (half-maximal effective dose) value for cisplatin (5.48 μg/mL vs. 61.76 μg/mL) and osimertinib (5.04 μM vs. 17.02 μM) was distinctly increased in resistant cells compared to treatment-naïve cells, confirming acquired resistance in UKF-PeC3^r^CIS^2^ and UKF-PeC3^r^OSI^2^ (Figure 3).

### 3.6. Expression of Protein Candidates in Cell Lines

**Figure 4:**
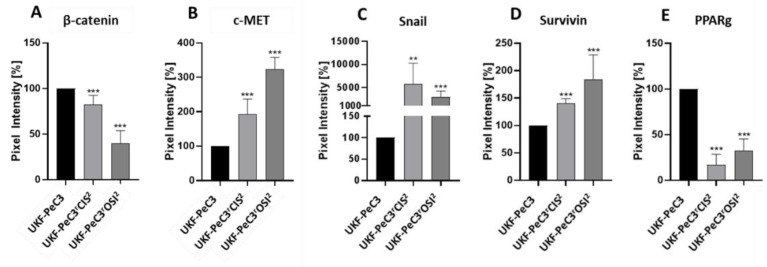
Western blot analysis: Pixel density analysis of the protein expression of β-catenin (**A**), c-Met (**B**), snail (**C**), survivin (**D**), and PPARg (**E**) in UKF-PeC3^r^CIS^2^ and PeC3^r^OSI^2^ cells, compared to UKF-PeC3 cells (set to 100%). Analysis of pixel density was normalized by a total protein staining. Error bars indicate the standard deviation (SD). Significant difference from UKF-PeC3: ** = *p* ≤ 0.01, *** = *p* ≤ 0.001.

In order to validate the TMA result in in vitro studies, protein expression profiles were assessed in therapy-naïve vs. resistant cell lines. In Western blot analysis, c-MET, snail, and survivin expressions were significantly increased in both resistant cell lines compared to the therapy-naïve cell line. Interestingly, ß-catenin expression was significantly decreased in both resistant cell lines compared to the parental cell line (Figure 4). On the contrary, n-myc was not detected in PeCa cell lines.

### 3.7. Modulation of Tumor Cell Growth by c-MET Inhibition

**Figure 5:**
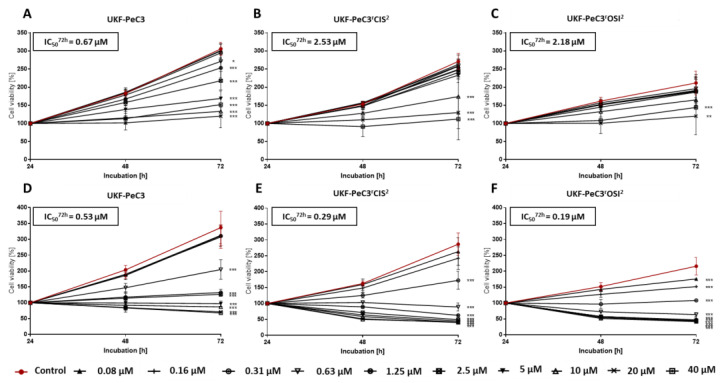
Tumor growth after exposure to cabozantinib (**A**–**C**) or tivantinib (**D**–**F**): Tumor cell viability of UKF-PeC3 (**A**,**C**), UKF-PeC3^r^CIS^2^ (**B**,**E**), and PeC3^r^OSI^2^ (**C**,**F**) cells after 24, 48, and 72 h treatment with ascending cabozantinib or tivantinib concentrations (0.08–40 µM). The cell number was set to 100% after 24 h incubation. The IC50 of cabozantinib and tivantinib after 72 h treatment is specified. Error bars indicate the standard deviation (SD). Significant difference from untreated control: * = *p* ≤ 0.05, ** = *p* ≤ 0.01, *** = *p* ≤ 0.001.

For the purpose of functional validation, c-MET blockage of PeCa UKF-PeC3 and resistant cell lines was performed by blocking studies with the highly selective c-MET inhibitor tivantinib and multi-kinase inhibitor cabozantinib. Drugs were administered in the range of 0.08 µM to 40 µM. Dose- and time-dependent growth inhibition was detected in the parental cell line and both resistant cell lines in comparison to their untreated controls (Figure 5).

A significant growth reduction was detected in all PeCa cell lines after 72 h treatment. Thereby, the effect of tivantinib was clearly stronger in both resistant cell lines since the IC50 value for tivantinib (0.53 µM in UKF-PeC3 vs. 0.29 µM in UKF-PeC3^r^CIS^2^ and 0.19 µM in UKF-PeC3^r^OSI^2^) was decreased significantly in resistant cells compared to treatment-naïve cells. In contrast, cabozantinib had a higher impact on tumor cell growth in the parental cell line UKF-PeC3 compared to the resistant cell lines since the IC50 value for cabozantinib (0.67 µM in UKF-PeC3 vs. 2.53 µM in UKF-PeC3^r^CIS^2^ and 2.18 µM in UKF-PeC3^r^OSI^2^) was decreased in UKF-PeC3 compared to the resistant cell lines. The trypan blue exclusion test did not show signs of toxicity.

## 4. Discussion

The designation of novel molecular targets is a crucial prerequisite for the establishment of efficacious treatment concepts fostering outcome optimization in men with advanced PeCa. In the current analysis, we focused on the role of selected molecular candidates related to c-MET signaling in tumor tissue to predict histopathologic and oncologic outcomes and the effect of their inhibition in unique treatment-naïve as well as resistant PeCa cell lines generated by our research group, thus mimicking clinical scenarios of the second- and third-line systemic treatment.

Out of six assessed candidate molecules, c-MET, a receptor tyrosine kinase encoded by the MET gene, turned out to be the most seminal target, providing a plausible red thread between both tumor tissue and cell lines. This transmembrane protein is a proficient oncogenic driver of cell proliferation, invasive growth, and epithelial-to-mesenchymal transition (EMT) [23]. cMET activates inter alia the STAT, PI3K, RAS, and MAPK pathways either through direct phosphorylation or by binding to other surface receptors in a membrane-spanning interaction [23]. Notably, stimulation of c-MET by hepatocyte growth factor (HGF) promotes nuclear translocation of β-catenin, which participates in transcriptional gene regulation [24]. Strikingly, a recent study by de Vries and coworkers demonstrated an at least 70% expression of c-MET in 14 out of 15 men with PeCa and proved the feasibility of fluorescence imaging with the c-MET targeting tracer EMI-137 for intraoperative tumor visualization using a cyanine-5 fluorescence camera [25]. In our analysis, c-MET was detectable in 95.7% of samples, whereas augmented expression of c-MET in tumor tissue was significantly associated with and independently predicted for unfavorable DSS. Interestingly, Gunia et al. did not find an association between positivity of the c-MET staining (87% of samples) and DSS either by the log-rank test or in multivariable Cox regression analysis in the investigation of 92 males with PeCa [26]. Importantly, the researchers stratified the cohort by the presence or absence of c-MET expression, while the optimal survival-based cut-off for classification of the c-MET expression as low or high was used in our study, reflecting a completely different scientific issue and resulting outcomes. In concert with our finding, Szturz and collaborators showed an association between a high c-MET expression and worse DSS, progression-free survival, and OS in their systematic review and meta-analysis of 2019 cases of squamous cell carcinoma of the head and neck [27], which is biologically somewhat related to PeCa [28]. Similarly, Ren and co-authors reported shorter DSS and OS in patients with c-MET overexpression in squamous cell carcinoma and adenocarcinoma of the esophagus [29].

Validating our observation in human tissue, c-MET expression was detected in both treatment-naïve and resistant PeCa cells, whereas its level was significantly elevated in both cisplatin- and osimertinib-resistant cell lines. This effect was the most pronounced one among all candidate molecules. While cisplatin is a backbone of chemotherapeutic regimens for the management of metastatic PeCa [30], no standard care protocols are defined for men upon progression after cytotoxic therapy. Given the frequently observed increased expression of EGFR, anti-EGFR inhibitors have been proposed as salvage treatment after failure of first-line chemotherapy for advanced PeCa, yielding a response rate of roughly 50% and PFS of 3 months [31,32,33]. Osimertinib is a third-generation EGFR tyrosine kinase inhibitor approved for metastatic EGFR-mutant non-small cell lung cancer [34], hence representing a possible option to treat PeCa in the second-line setting as a concept of drug repurposing.

Corroborating the findings in our cisplatin-resistant cell line, increased expression of c-MET has been reported to be associated with resistance to chemotherapy as well as EGFR inhibitors. Li and collaborators described an elevated expression of c-MET in cisplatin-resistant cell lines as compared to parental cell lines of ovarian cancer cells [35]. Blockade of c-MET promoted a reduction in cell growth and proliferation in their analysis. Similarly, Zhu et al. reported on the augmented expression of c-MET in non-small cell lung cancer cell lines with osimertinib resistance as compared to sensitive ones [36]. Treating resistant cells with the c-MET inhibitor capmatinib reduced EMT and self-renewal ability as well as promoting cell re-sensitization to osimertinib.

Strikingly, we observed an inverse trend of response to cell treatment with the multi-kinase inhibitor cabozantinib, approved for the therapy of advanced medullary thyroid cancer and hepatocellular and renal cell carcinoma [37], and the experimental c-MET inhibitor tivantinib comparing resistant and parental cells. Indeed, lower doses of cabozantinib were required in treatment-naïve than in resistant cells in order to achieve the same viability suppression. This finding supports the idea of testing cabozantinib as the initial step of systemic disease management, as it is now being assessed in the ongoing CaboPen trial [38]. Conversely, a higher sensitivity to tivantinib could be detected both in cisplatin- and osimertinib-resistant cells, pointing to the promising option of utilizing this agent in subsequent treatment lines in advanced PeCa. It is likely that an alternate mechanism of action of tivantinib might have contributed to this observation. Although initially thought to be a selective MET inhibitor [39], the cell cytoskeleton proteins vinculin and RhoC in melanoma cells and Glycogen synthase kinase 3α/β (GSK3α/β) as a pro-tumorigenic signaling protein relevant in acute myeloid leukemia (AML) and lung cancer cells have been identified as molecular targets of tivantinib as well [40,41,42]. Besides c-MET, interference with other elements which are essential for resistance to cisplatin and osimertinib might therefore explain the diverging effects in the dose-dependent response between cabozantinib and tivantinib in relation to parental and resistant cells in our study. Further profound assessment of this complex molecular circuitry is warranted to elucidate the observed difference between compounds.

Of note, resistant cell lines exhibited diminished expression of β-catenin corresponding to its reduced staining in the tumor tissue of a higher grade in our assessment. This is in accordance with an observed decrease in the number of β-catenin-positive cases and the intensity of expression in parallel with a reduction in tumor differentiation in an immunohistochemical study on oral squamous cell carcinoma [43]. Notably, recent evidence has highlighted the tumor-suppressing properties of β-catenin, which might explain the aforementioned finding of its inverse association with tumor grade and drug resistance [44]. Nevertheless, the exact role of β-catenin in cancer development and progression remains to be elaborated, since an association between β-catenin pathway activation and drug resistance as well as inferior outcomes has also been reported for some malignancies [12,45,46]. Importantly, β-catenin was neither predictive of survival nor of histopathological results in our study.

Snail, a transcriptional repressor controlling EMT, and survivin, a member of the inhibitor of apoptosis protein family, have both been shown to be activated and correlate with tumor grade, nodal metastasis, unfavorable outcomes, and drug resistance in different metastasized tumors [13,47,48]. In concert with these data, both candidate molecules were upregulated to different extents in resistant cell lines as compared to parental ones in our investigation. However, we have not identified a reliable association between their respective expression levels in the tumor tissue and patient oncological characteristics.

Ambivalent evidence exists on the role of PPARg, a ligand-dependent transcription factor, in tumorigenesis. While its oncogenic effects on tumor development and progression have been described in prostate cancer, studies on colon, esophageal, lung, and head and neck cancers display its antiproliferative and proapoptotic characteristics [49,50]. In accordance with these tumor-suppressive properties, we observed downregulation of PPARg in treatment-resistant as compared to naïve cells, whereas neither potential for survival prediction nor an association with histopathological findings was noted. Further scientific efforts are desirable to more precisely define the value of PPARg in the tumor-related context.

Owing to the rarity of PeCa, the main shortcoming of our work is the restricted number of cell lines as well as the sample size curtailing the validity of statistical models and hence the generalization of the findings. Moreover, pathological information on lymphovascular invasion and the resulting discrimination of pT1 in pT1a and pT1b disease was first available from 2010, thus ruling out inclusion of these variables in statistical modeling due to the missing values, as previously described [11,51,52]. Notwithstanding these flaws, we believe to have delivered a relevant building block for the future groundwork of targeted therapy in advanced PeCa.

## 5. Conclusions

In our study, c-MET was considered the most promising biomarker. This protein might offer an intriguing approach as a prognostic or therapeutic target, thus enabling novel therapeutic options for PeCa patients.

## Figures and Tables

**Table 1:** Clinicopathological features of patients.

	Total Cohort (n = 94)
Age at diagnosis	
Mean (SD)	64.9 (11.8)
Median [Min, Max]	67.0 [31.0, 90.0]
≤65	45 (47.9%)
>65	49 (52.1%)
Primary surgery	
Circumcision	8 (8.5%)
Tumor excision	5 (5.3%)
Partial penectomy	55 (58.5%)
Total penectomy	25 (26.6%)
Missing	1 (1.1%)
Tumor grade	
Low (G1/G2)	70 (74.5%)
High (G3/G4)	22 (23.4%)
Missing	2 (2.1%)
pT stage	
pT1	36 (38.3%)
pT2	30 (31.9%)
pT3	24 (25.5%)
Missing	4 (4.3%)
HPV status	
Negative	65 (69.1%)
Positive	24 (25.5%)
Missing	5 (5.3%)
pN stage	
NX-0	77 (81.9%)
N1	4 (4.3%)
N2	8 (8.5%)
N3	5 (5.3%)
Recurrence status	
No	66 (70.2%)
Yes	28 (29.8%)
Recurrence site	
None	66 (70.2%)
Local	18 (19.2%)
Systemic	10 (10.6%)
Adjuvant therapy	
None	68 (72.3%)
CTX	16 (17.0%)
Radiation	1 (1.1%)
CTX and Radiation	2 (2.1%)
Missing	7 (7.4%)
Tumor-dependent death	
No	61 (64.9%)
Yes	12 (12.8%)
Missing	21 (22.3%)

**Table 2:** Multivariate COX regression analysis of OS, DSS, and RFS.

Characteristic	OS		RFS		DSS	
	HR (95% CI)	*p*	HR (95% CI)	*p*	HR (95% CI)	*p*
PPARg						
Low expression	1.00 (reference)		1.00 (reference)		1.00 (reference)	
High expression	2.77 (0.74–10.36)	0.10	1.59 (0.41–6.13)	0.50	5.18 (0.95–28.32)	0.06
β-catenin						
Low expression	1.00 (reference)		1.00 (reference)		1.00 (reference)	
High expression	0.76 (0.30–1.89)	0.60	1.34 (0.52–3.40)	0.50	0.74 (0.16–3.42)	0.70
c-MET						
Low expression	1.00 (reference)		1.00 (reference)		1.00 (reference)	
High expression	1.38 (0.57–3.37)	0.50	1.05 (0.38–2.92)	0.90	5.03 (1.08 -23.32)	0.04
Grading						
low (G1/G2)	1.00 (reference)		1.00 (reference)		1.00 (reference)	
high (G1/G2)	1.72 (0.72–4.10)	0.20	1.45 (0.48–4.32)	0.50	0.40 (0.08–2.13)	0.28
T-Stage						
pT1/pT2	1.00 (reference)		1.00 (reference)		1.00 (reference)	
pT3	0.89 (0.34–2.33)	0.80	0.73 (0.27–1.97)	0.50	0.56 (0.11–2.85)	0.48
N-Stage						
N0	1.00 (reference)		1.00 (reference)		1.00 (reference)	
N1/N2/N3	1.84 (0.67–5.04)	0.20	1.11 (0.33–3.80)	0.90	14.09 (3.29–60.43)	<0.001

## Data Availability

All data generated or analyzed during this study are included in this published article and its supplementary information files.

## Supplementary Materials

The following supporting information can be downloaded at: https://www.mdpi.com/article/10.3390/cancers14071683/s1, Figure S1: Dichotomized biomarker expressions of β-catenin and survivin correlated with clinical and histopathological data (Fischer’s exact test). Higher survivin expression values were more common in patients with HPV infection and obesity (both *p* ≤ 0.05). β-catenin was inversely associated with tumor grading (*p* ≤ 0.05). Figure S2: Fischer’s exact test: c-MET and PPARg did not correlate with HPV infection. Figure S3: Biomarker correlations by Spearman‘s coefficient analysis. Spearman correlation coefficient (rs) depicted in the ovals (blue ovals = positive rs, red ovals = negative rs; the darker the color shade—the stronger the strength of association; non-scored-out ovals = significant association). Figure S4: Kaplan–Meier Analysis of clinical and histopathological data. Age at diagnosis (>65 years) was associated with a shortened RFS (*p* = 0.02) and node-positive disease (N1–N3) was associated with a decreased DSS (*p* = 0.001). Figure S5: Kaplan–Meier Analysis showed no association between HPV infection and survival. Supplemental Figure S6: Detailed information of Figure 4. Protein expressions in parental, cisplatin-resistant and osimertnib-resistant PeC3 cells. Protein expression of β-catenin, c-MET, snail, survivin and PPARg in UKF-PeC3, UKF-PeC3rCIS2 and PeC3rOSI2 (A), corresponding Coomassie blue staining of total protein (B). Table S1: Selection of cut-off scores (low and high) based on receiver operating characteristic (ROC) curve analysis; Table S2: Immunohistochemical staining results of biomarkers in tumor tissue.
