# Supplementary material for: Value of c-MET and Associated Signaling Elements for Predicting Outcomes and Targeted Therapy in Penile Cancer
Source: Cancers (Basel). 2022 Mar 25;14(7):1683. doi: 10.3390/cancers14071683 (PMC8997038; doi:10.3390/cancers14071683)

A PeC3 β-catenin 92 kDa

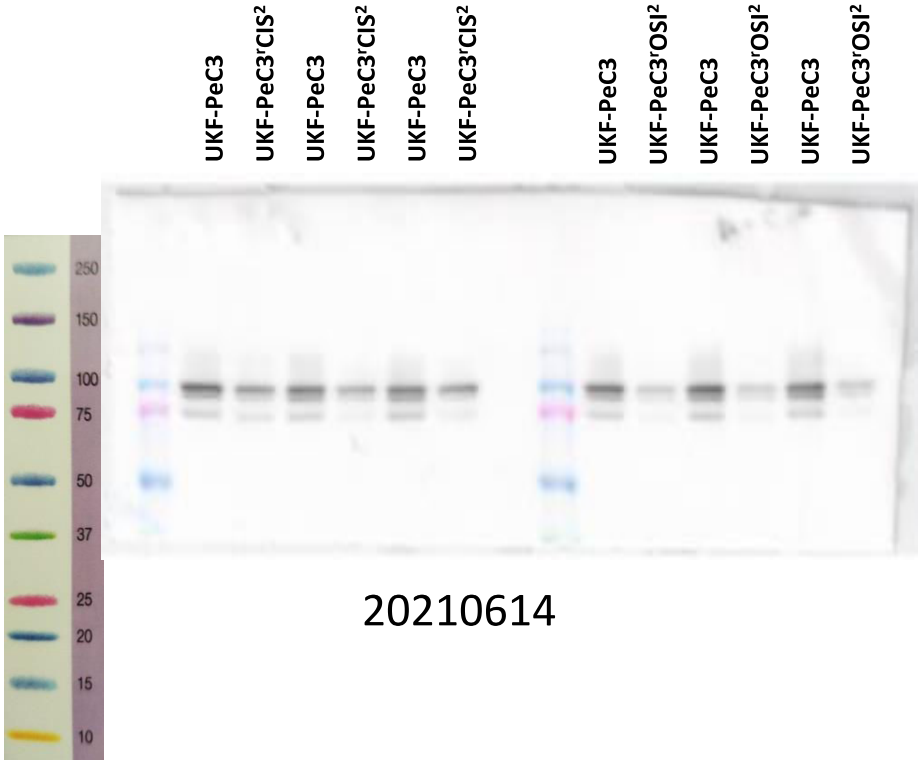

20210614

B Coomassie Brilliant Blue

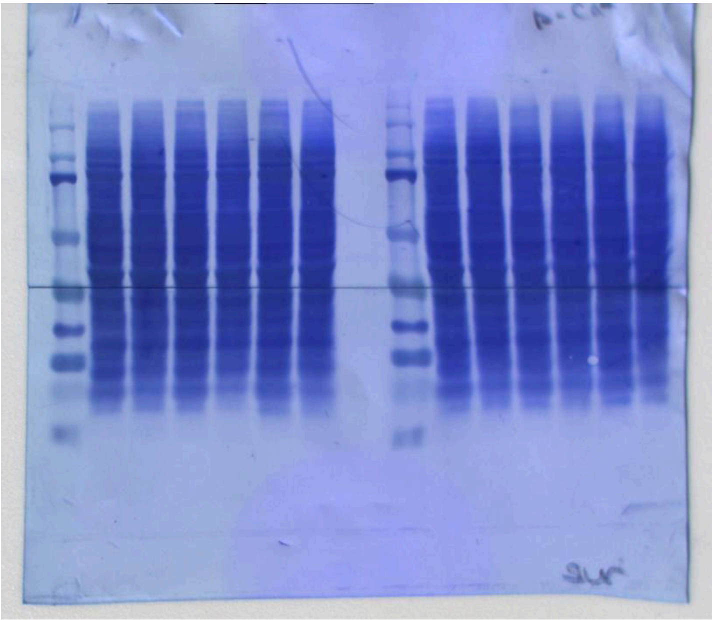

A PeC3 β-catenin 92 kDa

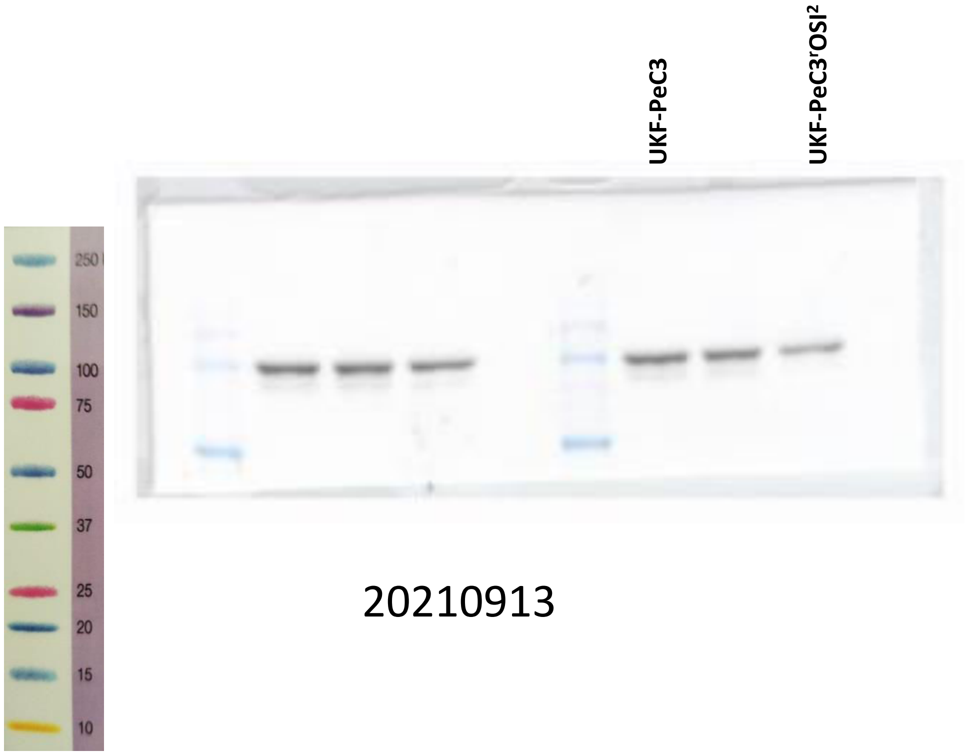

20210913

B Coomassie Brilliant Blue

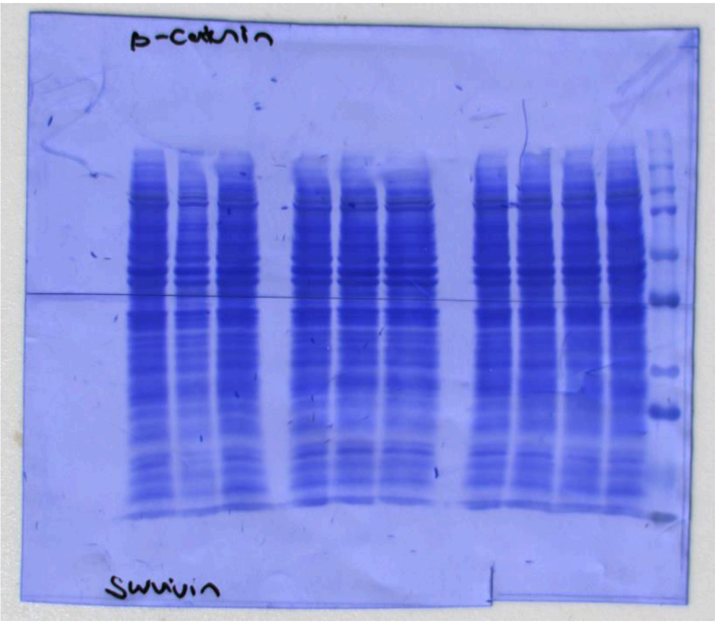

A PeC3 β-Catenin 92 kDa

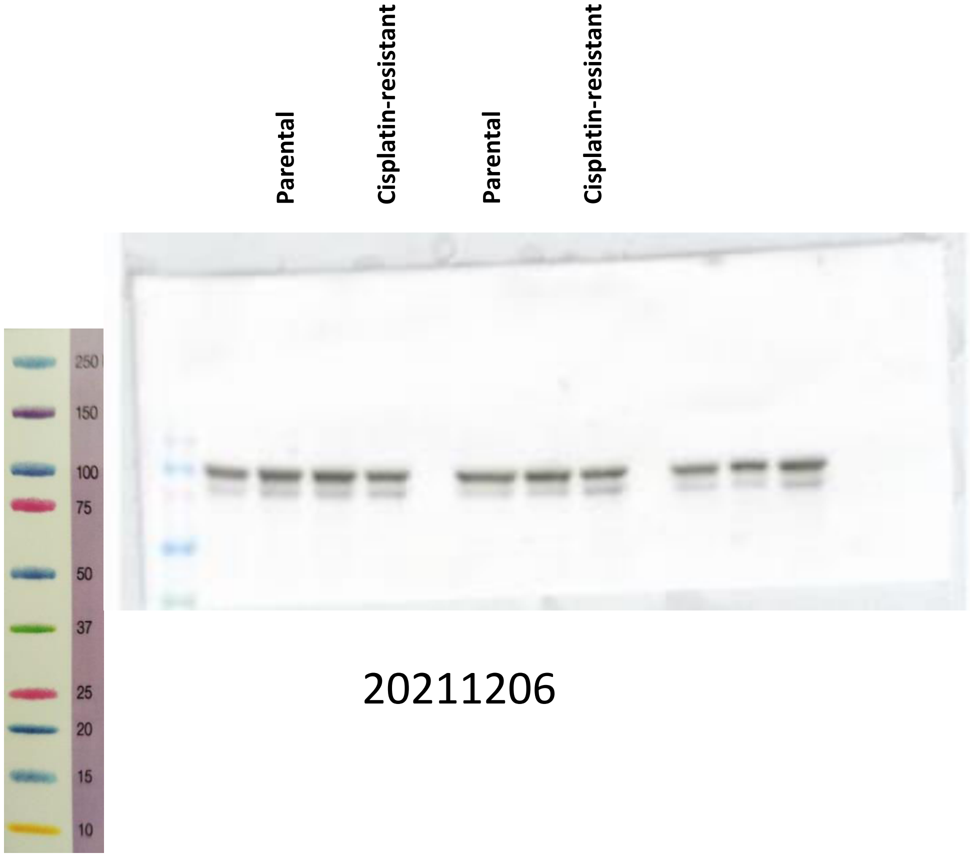

20211206

B Coomassie Brilliant Blue

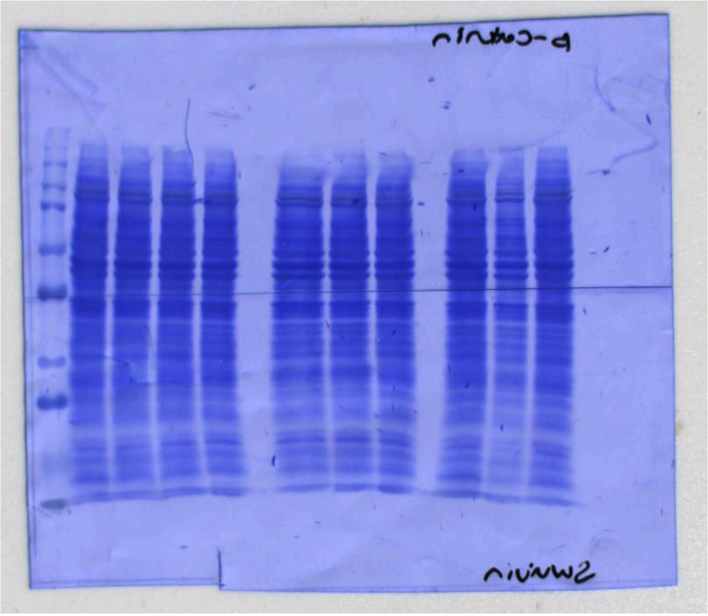

S7

A PeC3 c-MET 140 kDa

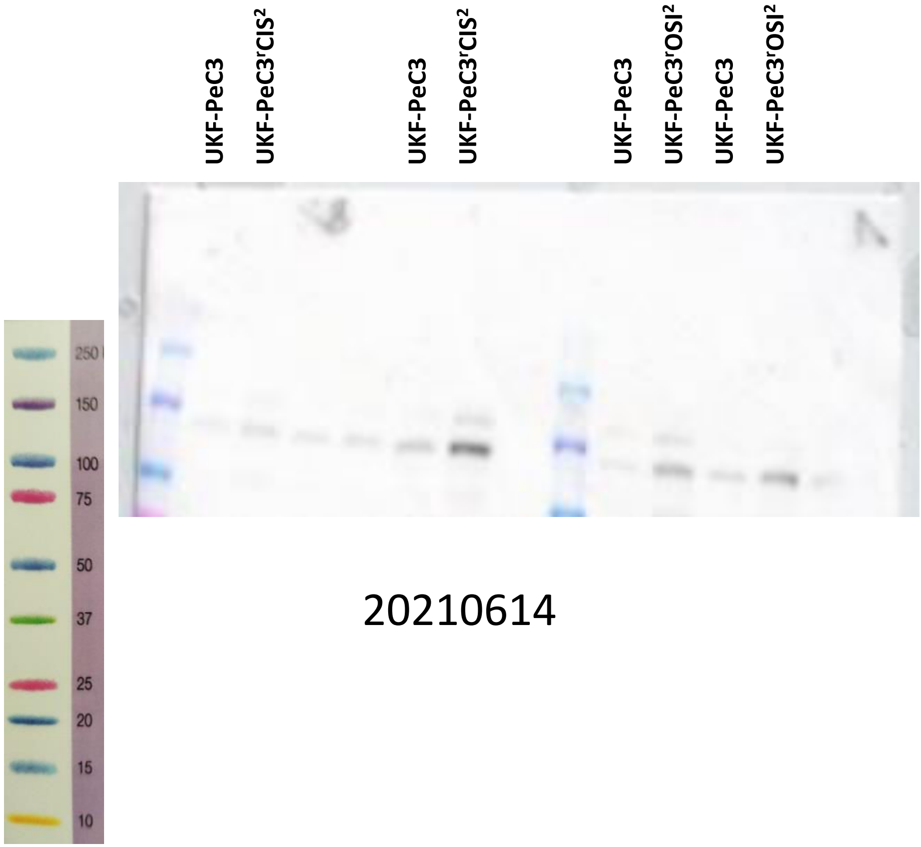

20210614

B Coomassie Brilliant Blue

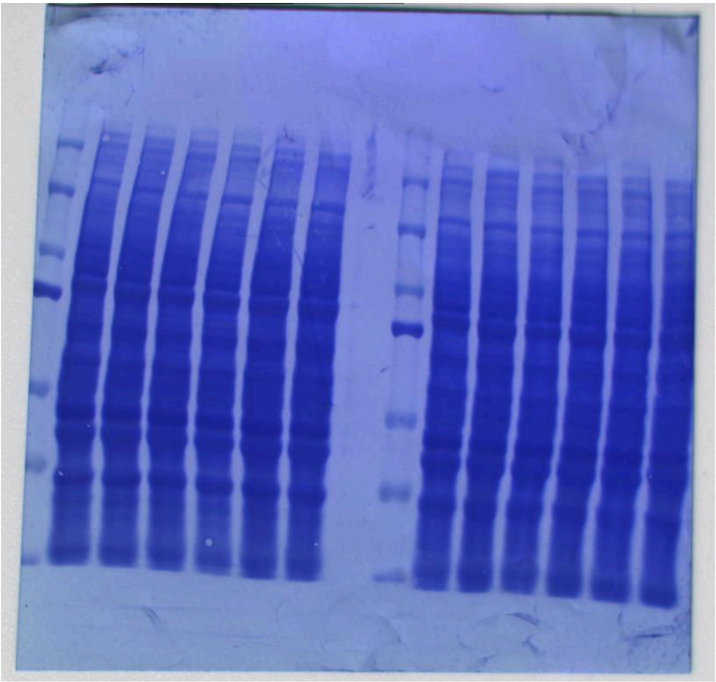

S8

A PeC3 c-MET 140 kDa

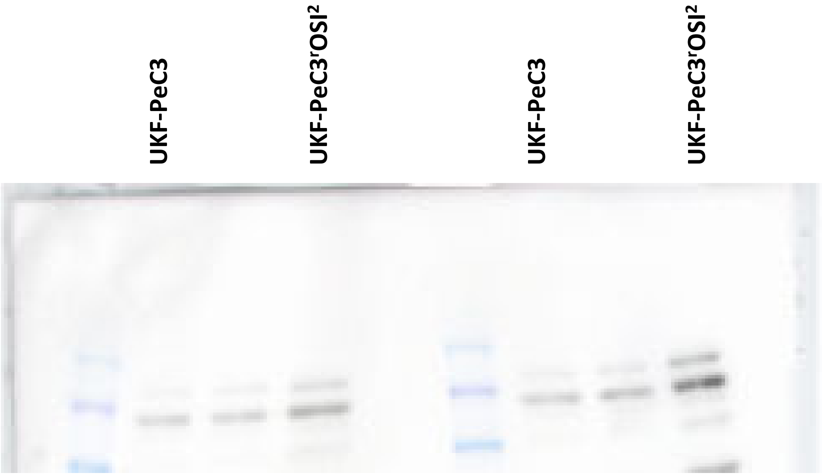

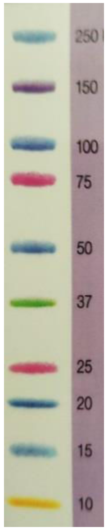

20210913

B Coomassie Brilliant Blue

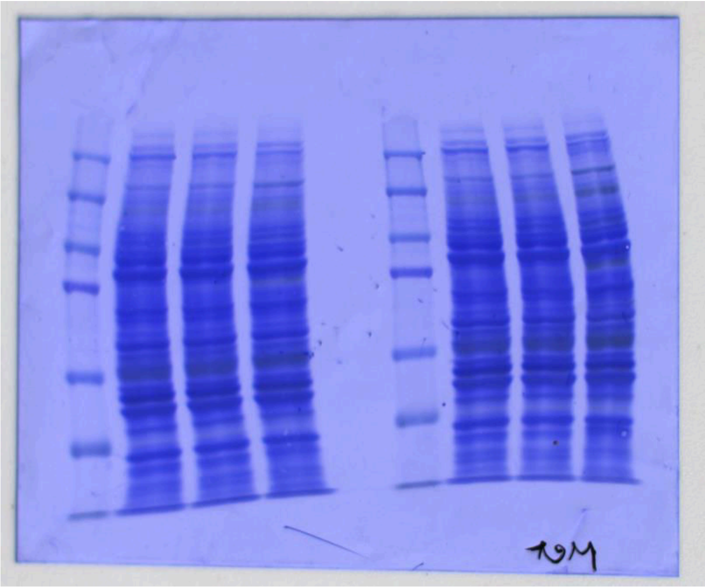

S9

A PeC3 c-MET 140 kDa

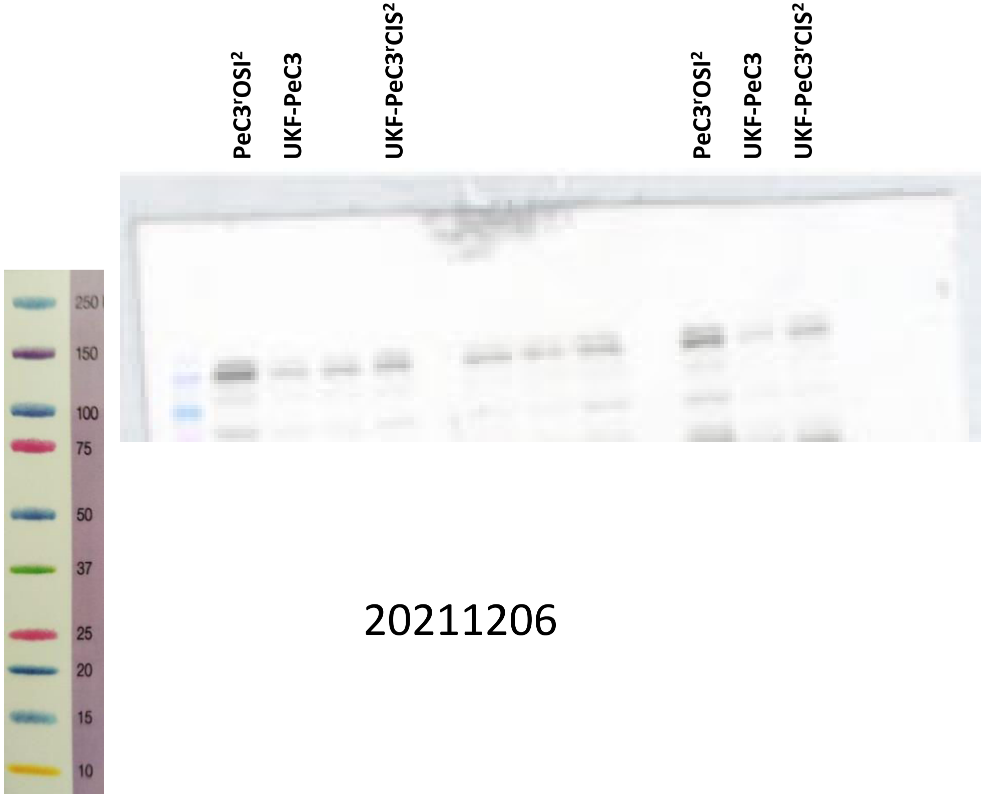

20211206

B Coomassie Brilliant Blue

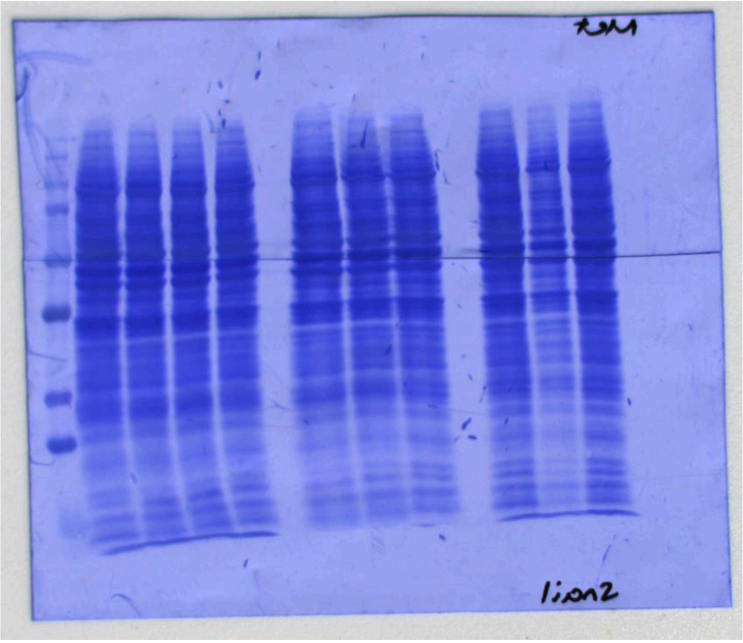

KW24 - Snail –Western Blot

A

PeC3 Snail 29 kDa

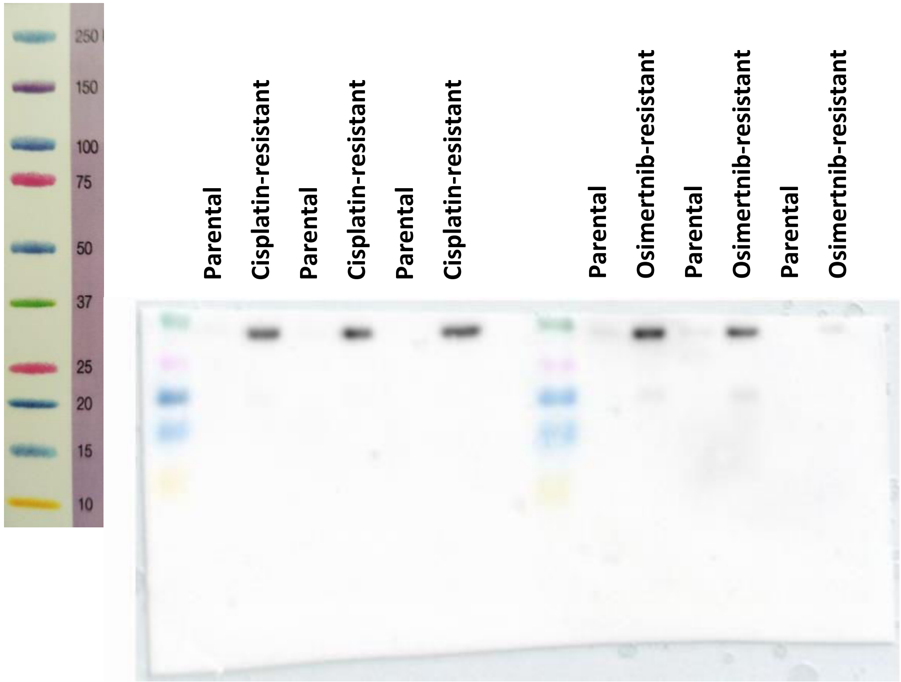

20210614

B

Coomassie Brilliant Blue

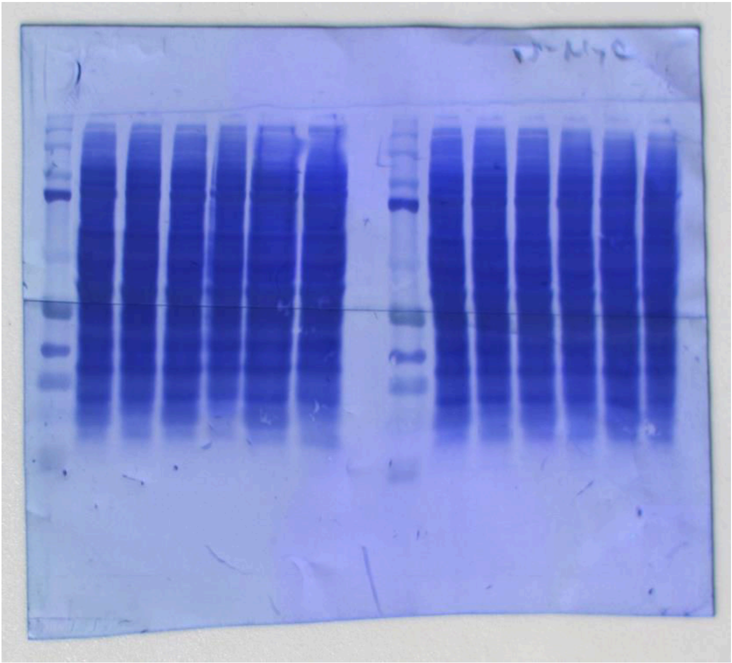

KW37 - Snail – Western Blot

A PeC3 Snail 29 kDa

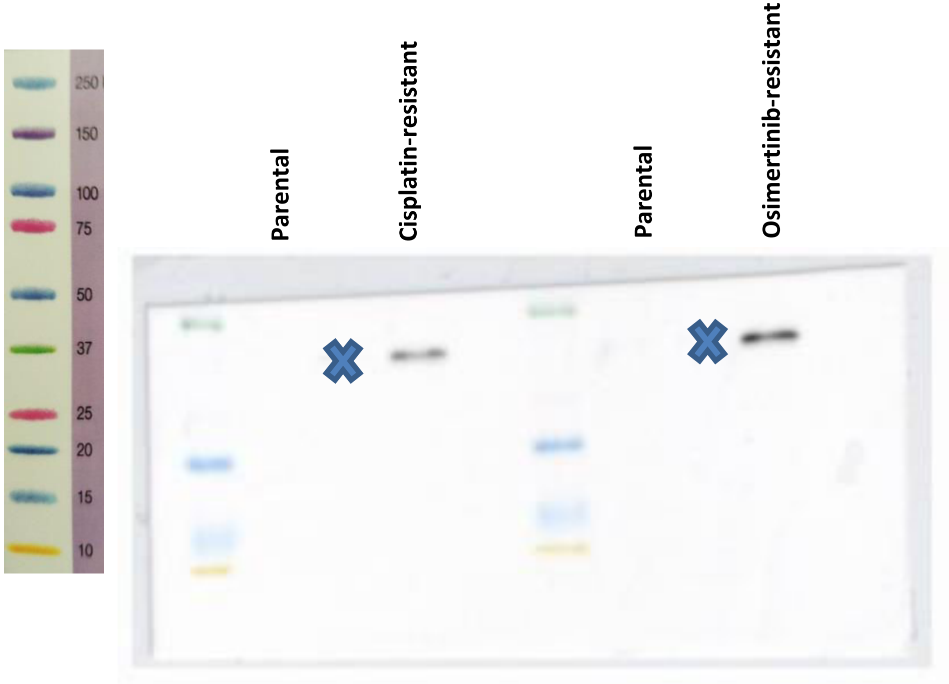

20210913

B Coomassie Brilliant Blue

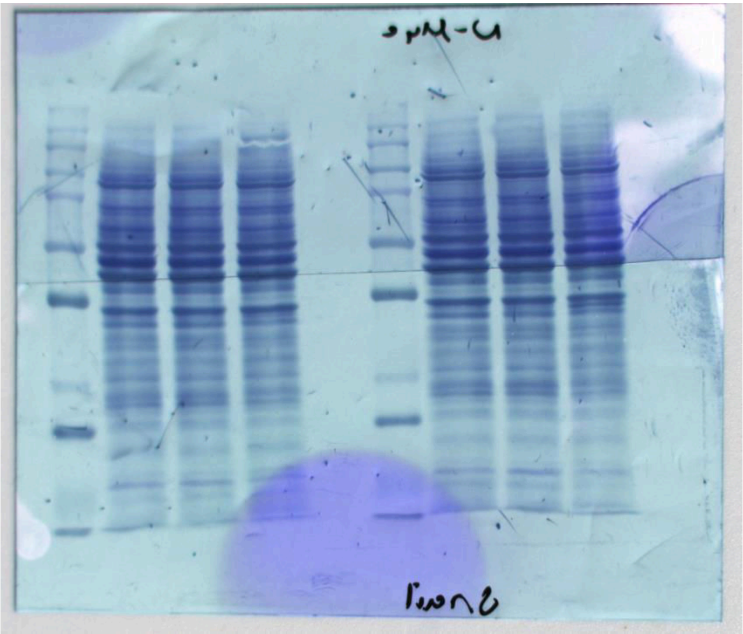

KW49 - Snail –Western Blot

A

PeC3 Snail 29 kDa

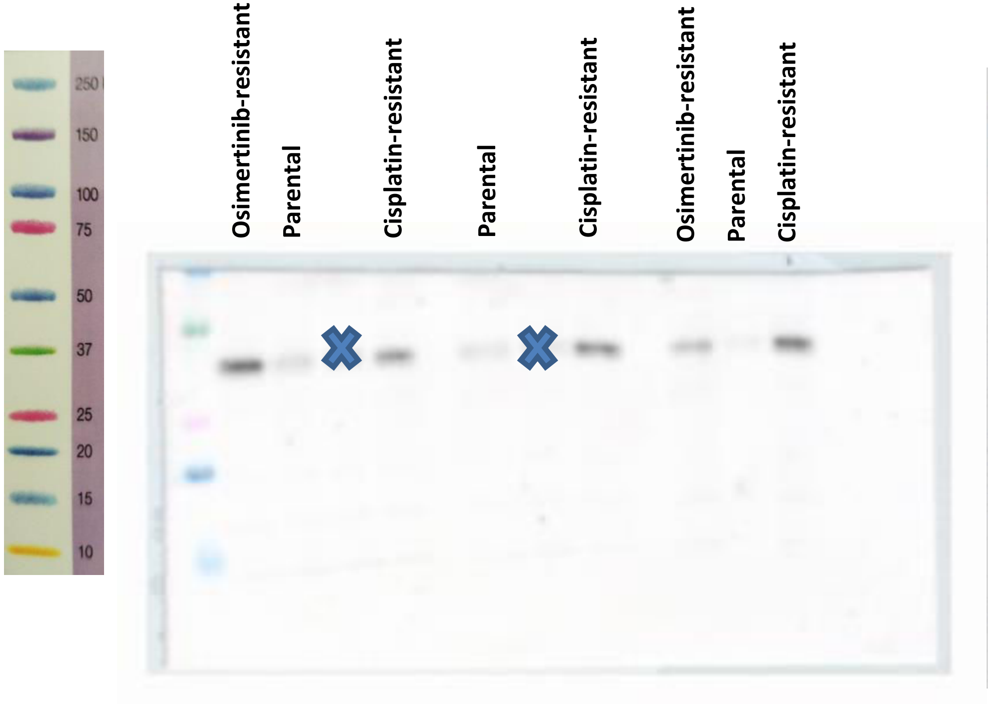

20211206

B

Coomassie Brilliant Blue

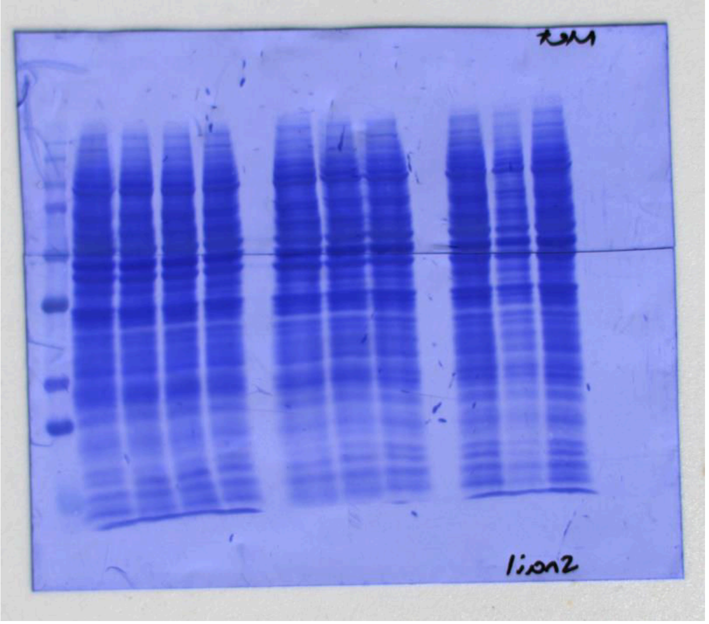

KW24 - Survivin –Western Blot

A

PeC3 Survivin 16 kDa

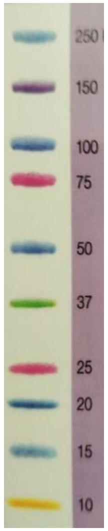

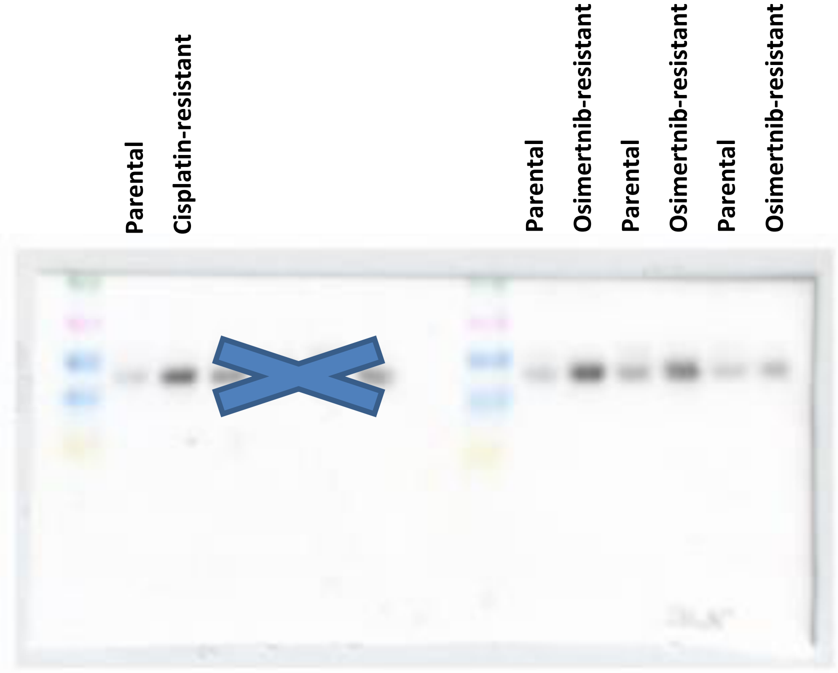

20210614

B

Coomassie Brilliant Blue

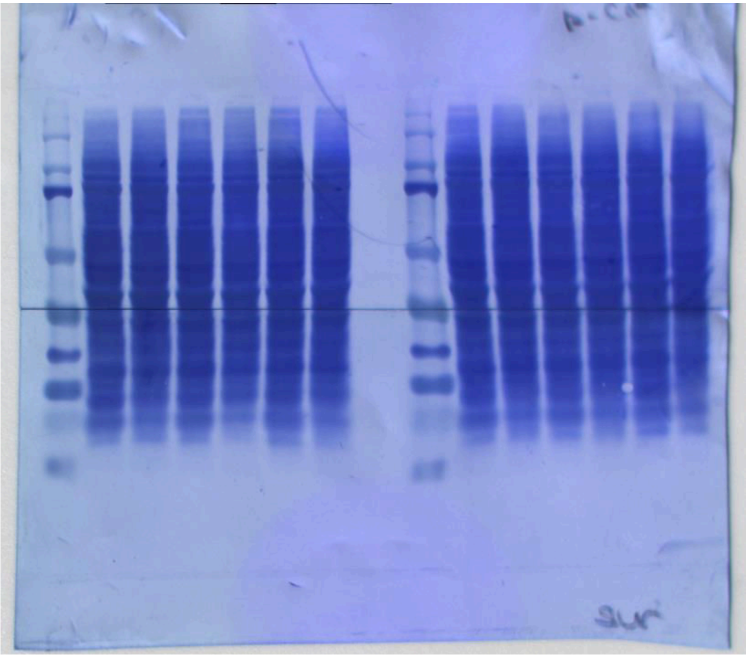

KW37 - Survivin – Western Blot

A PeC3 Survivin 16 kDa

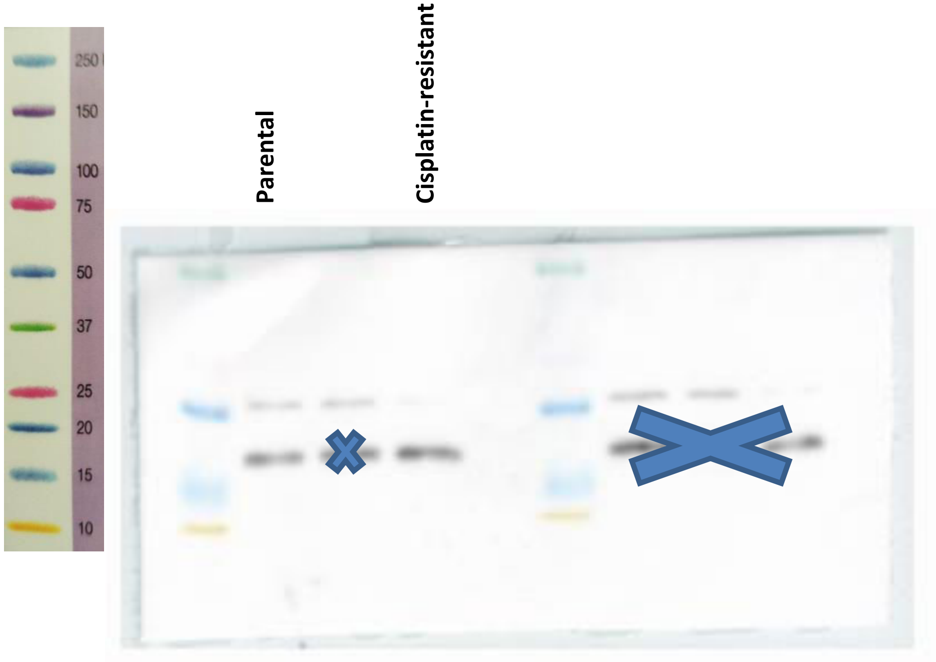

20210913

B Coomassie Brilliant Blue

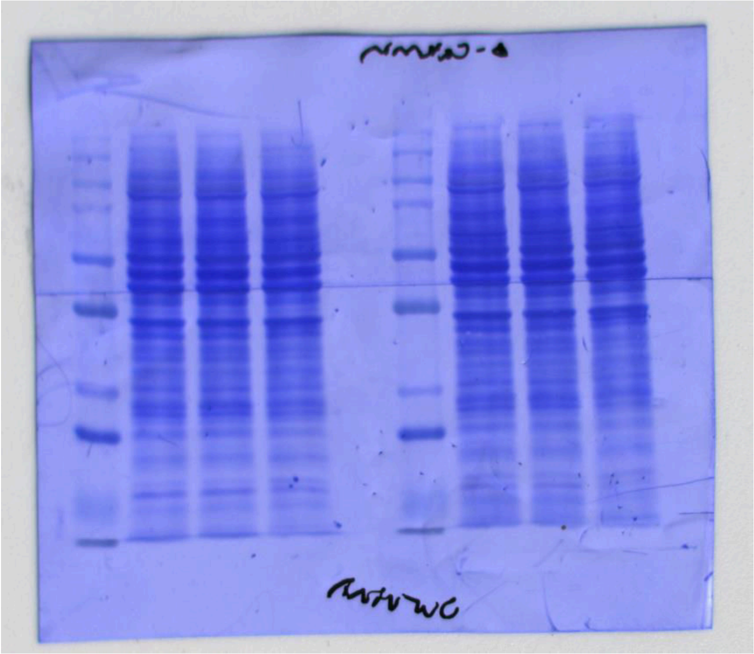

KW49 – Survivin –Western Blot

A

PeC3 Survivin 29 kDa

Osimertinib-resistant **Parental** **Cisplatin-resistant** **Parental** **Cisplatin-resistant** **Osimertinib-resistant** **Parental** **Cisplatin-resistant**

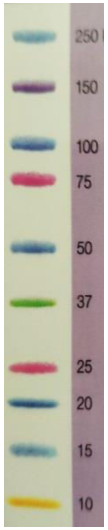

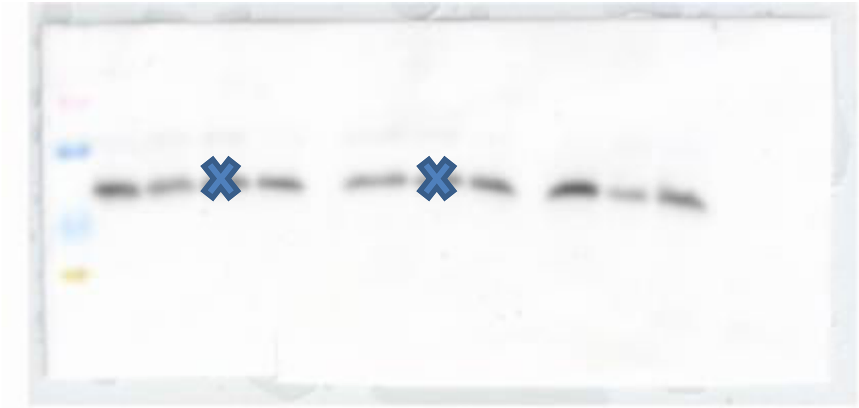

20211206

B

Coomassie Brilliant Blue

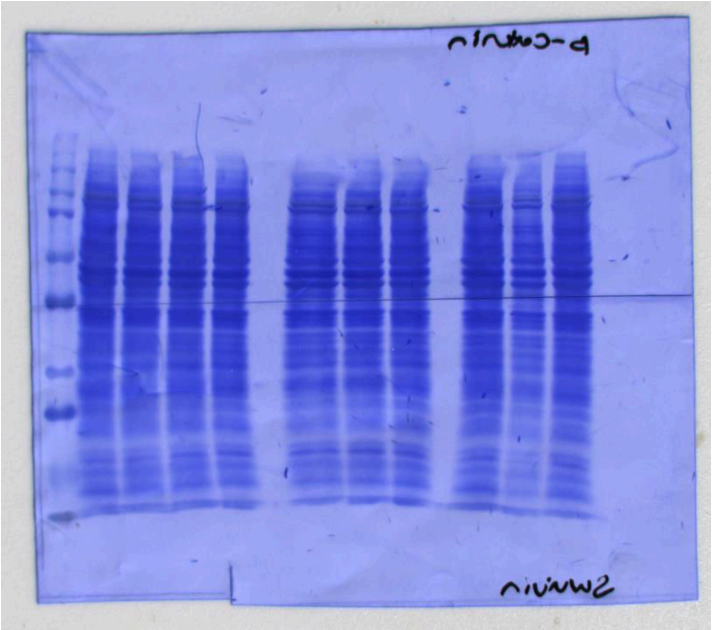

KW37 - PPARPγ –Western Blot

PeC3 PPARPγ 53,57 kDa

A

Parental | Osimertinib-resistant | Parental | Osimertinib-resistant

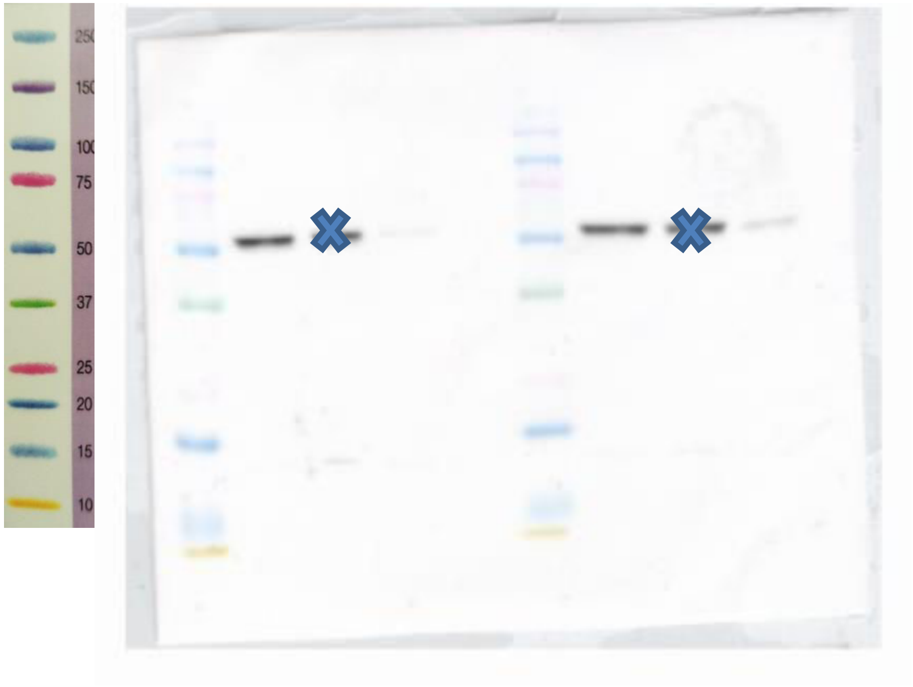

20210913

B

Coomassie Brilliant Blue

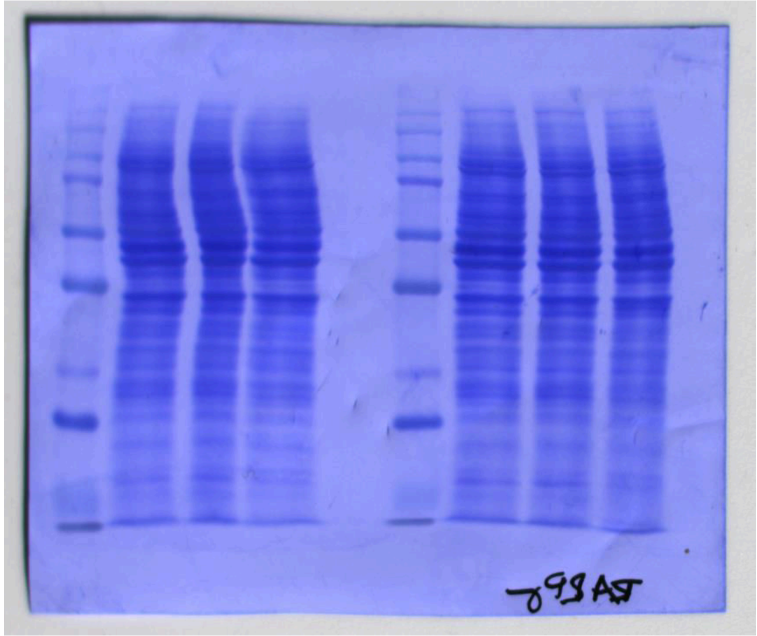

KW49 – PPARPγ –Western Blot

A

PeC3 PPARPγ 53,57 kDa

Osimertinib-resistant | **Parental** | **Cisplatin-resistant** | **Parental** | **Cisplatin-resistant** | **Osimertinib-resistant** | **Parental** | **Cisplatin-resistant**

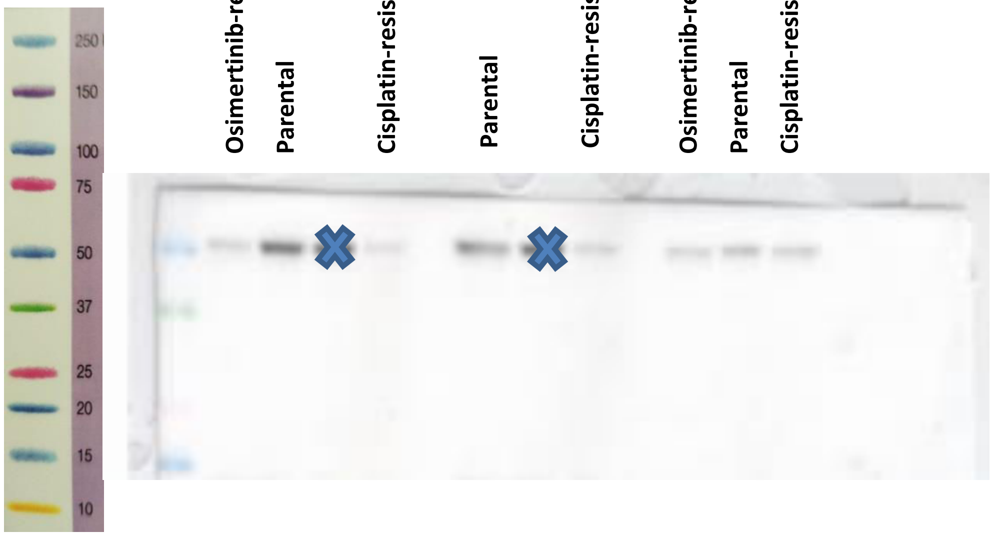

20211206

B

Coomassie Brilliant Blue

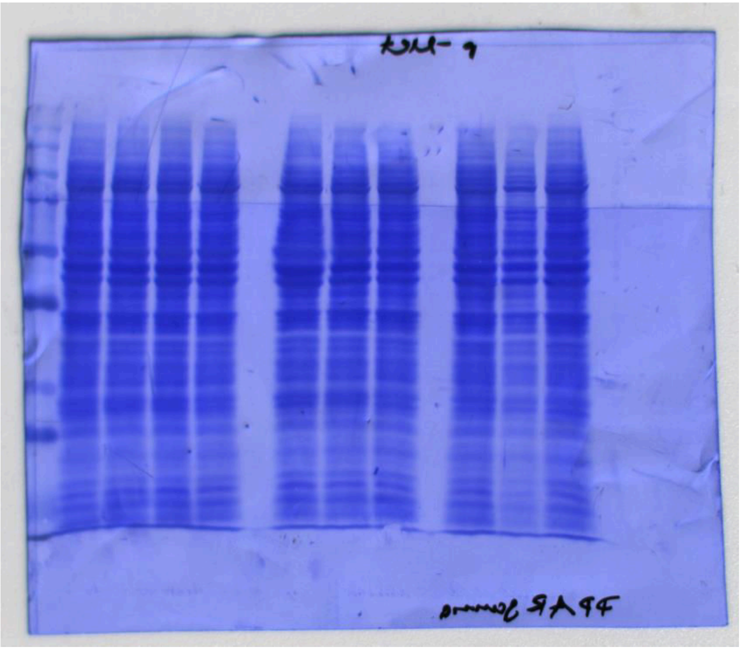

Supplement: Supplementary file 1 [file cancers-14-01683-s001.zip › Figure S6.pdf]
