# Supplementary material for: Value of c-MET and Associated Signaling Elements for Predicting Outcomes and Targeted Therapy in Penile Cancer
Source: Cancers (Basel). 2022 Mar 25;14(7):1683. doi: 10.3390/cancers14071683 (PMC8997038; doi:10.3390/cancers14071683)
Supplement: Supplementary file 1 [file cancers-14-01683-s001.zip › Table_S1.pdf]

Biomarker	value
Survivin_irs_os	11
PPARg_irs_os	0,25
β-Catenin_irs_os	0,25
Snail_irs_os	3,75
n-myc_irs_os	1,75
c-MET_irs_os	0,75
