# Supplementary material for: Value of c-MET and Associated Signaling Elements for Predicting Outcomes and Targeted Therapy in Penile Cancer
Source: Cancers (Basel). 2022 Mar 25;14(7):1683. doi: 10.3390/cancers14071683 (PMC8997038; doi:10.3390/cancers14071683)
Supplement: Supplementary file 1 [file cancers-14-01683-s001.zip › Table_S2.pdf]

	Overall (N=94)
Survivin	
low	68 (72.3%)
high	23 (24.5%)
Missing	3 (3.2%)
PPARg	
low	83 (88.3%)
high	9 (9.6%)
Missing	2 (2.1%)
β-Catenin	
low	49 (52.1%)
high	41 (43.6%)
Missing	4 (4.3%)
Snail	
low	87 (92.6%)
high	3 (3.2%)
Missing	4 (4.3%)
n-myc	
low	74 (78.7%)
high	16 (17.0%)
Missing	4 (4.3%)
c-MET	
low	49 (52.1%)
high	41 (43.6%)
Missing	4 (4.3%)
