# Supplementary figures and images for: Value of c-MET and Associated Signaling Elements for Predicting Outcomes and Targeted Therapy in Penile Cancer
Source: Cancers (Basel). 2022 Mar 25;14(7):1683. doi: 10.3390/cancers14071683 (PMC8997038; doi:10.3390/cancers14071683)

# Figure S1

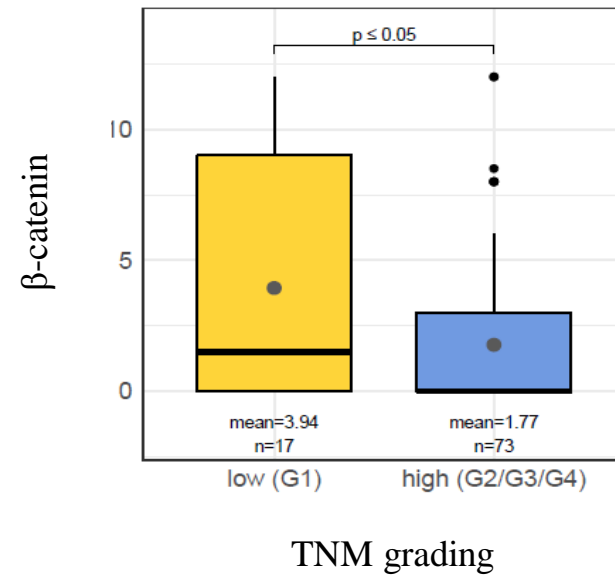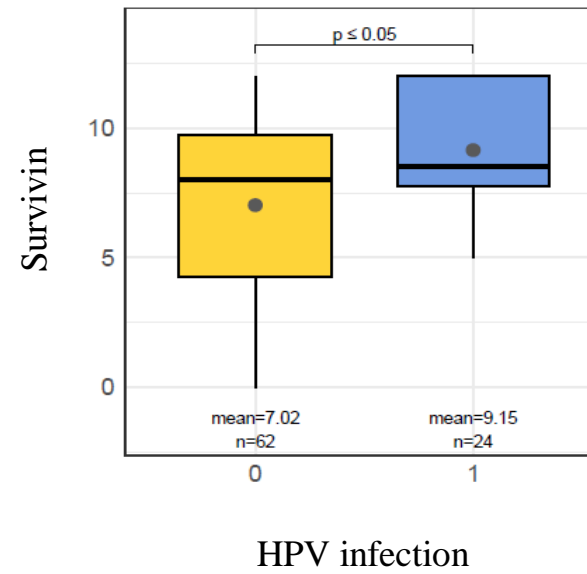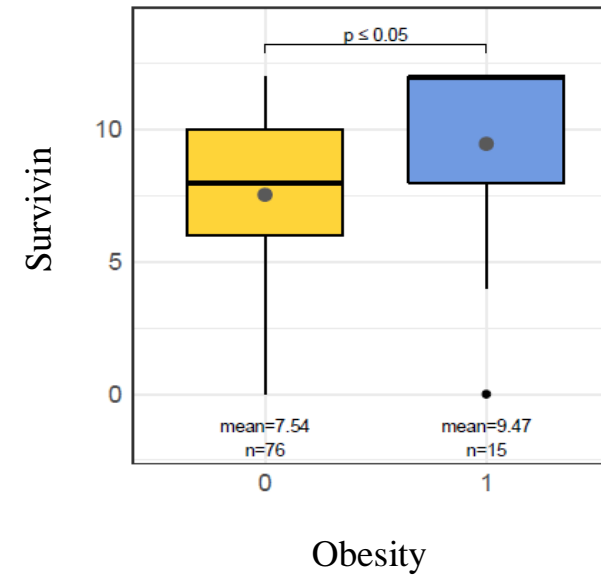

Supplement: Supplementary file 1 [file cancers-14-01683-s001.zip › Figure S1.pdf]

# Figure S2

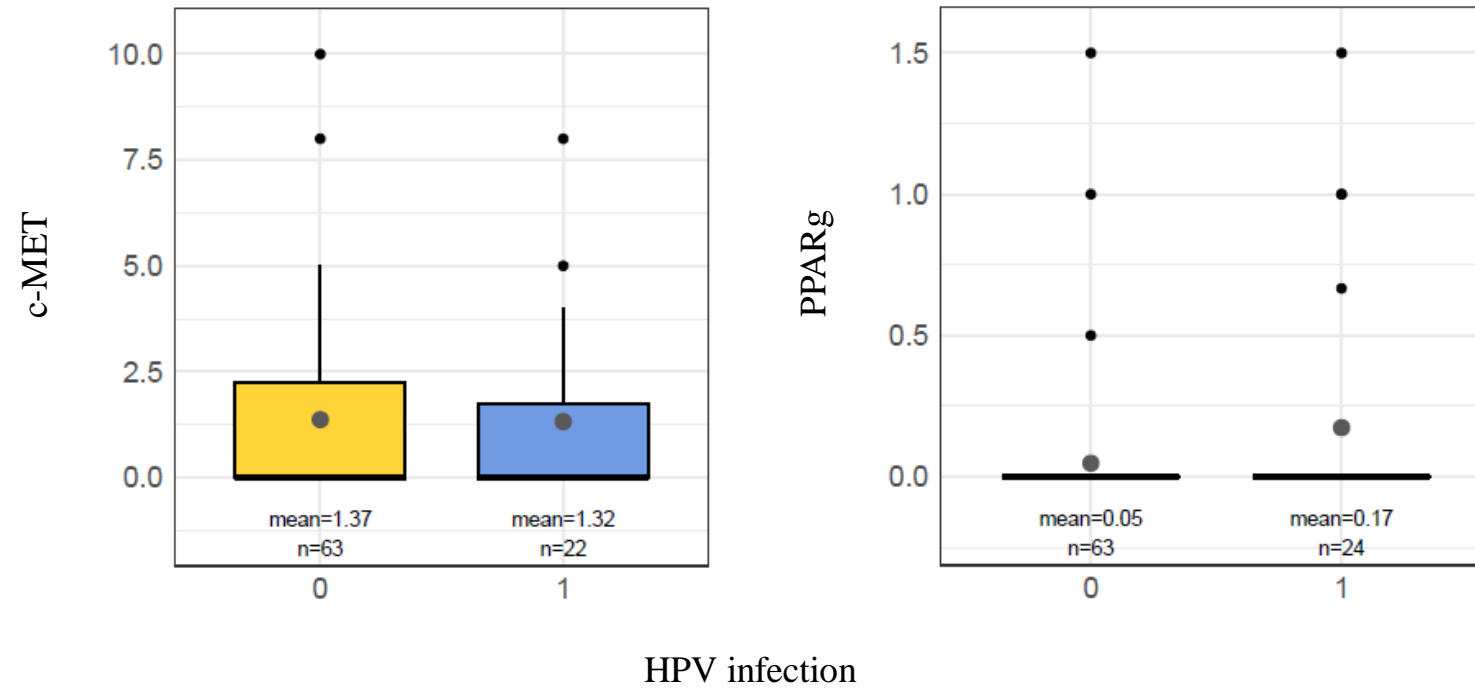

Supplement: Supplementary file 1 [file cancers-14-01683-s001.zip › Figure S2.pdf]

# Figure S3

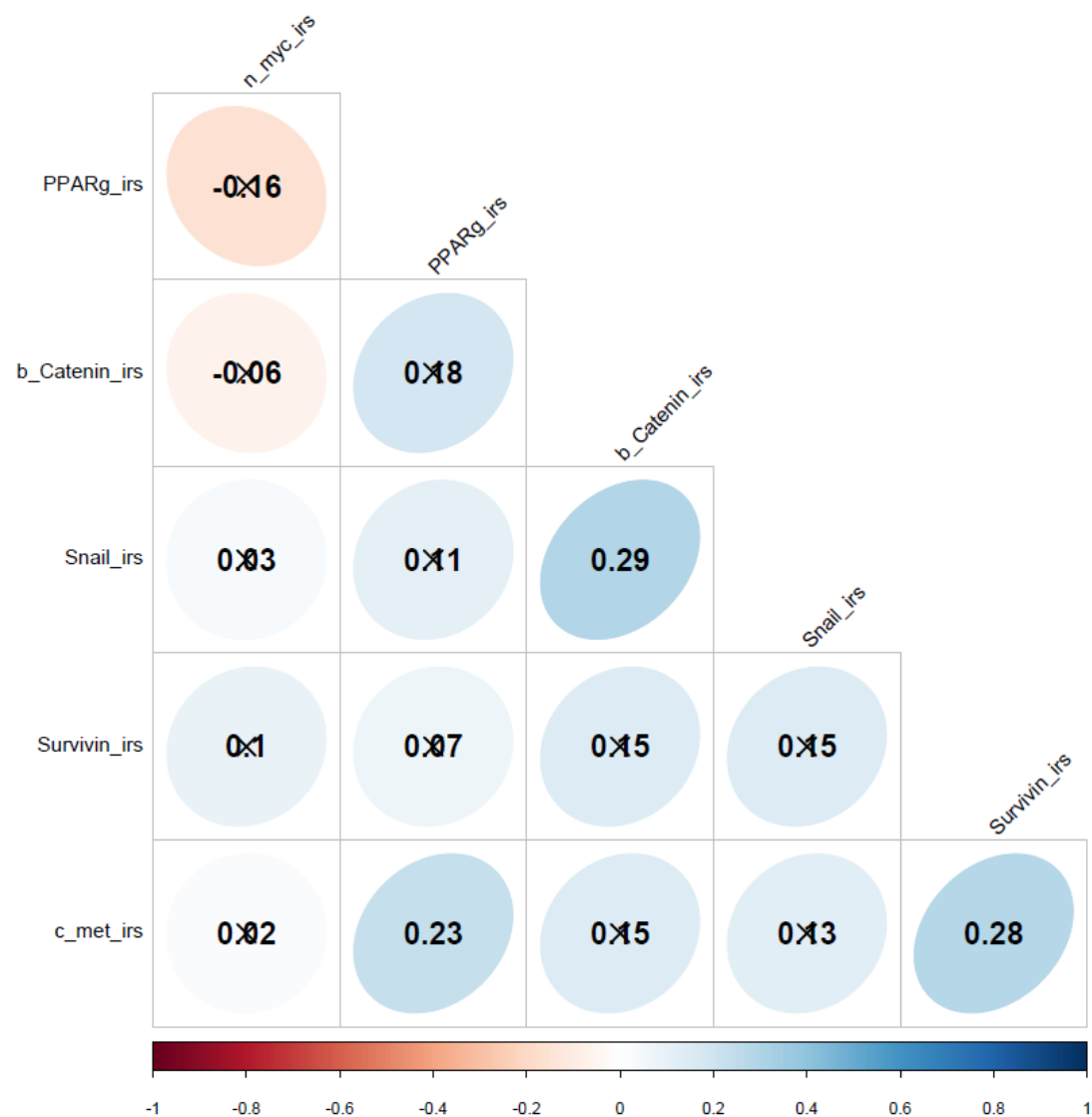

Supplement: Supplementary file 1 [file cancers-14-01683-s001.zip › Figure S3.pdf]

# Figure S4

Age

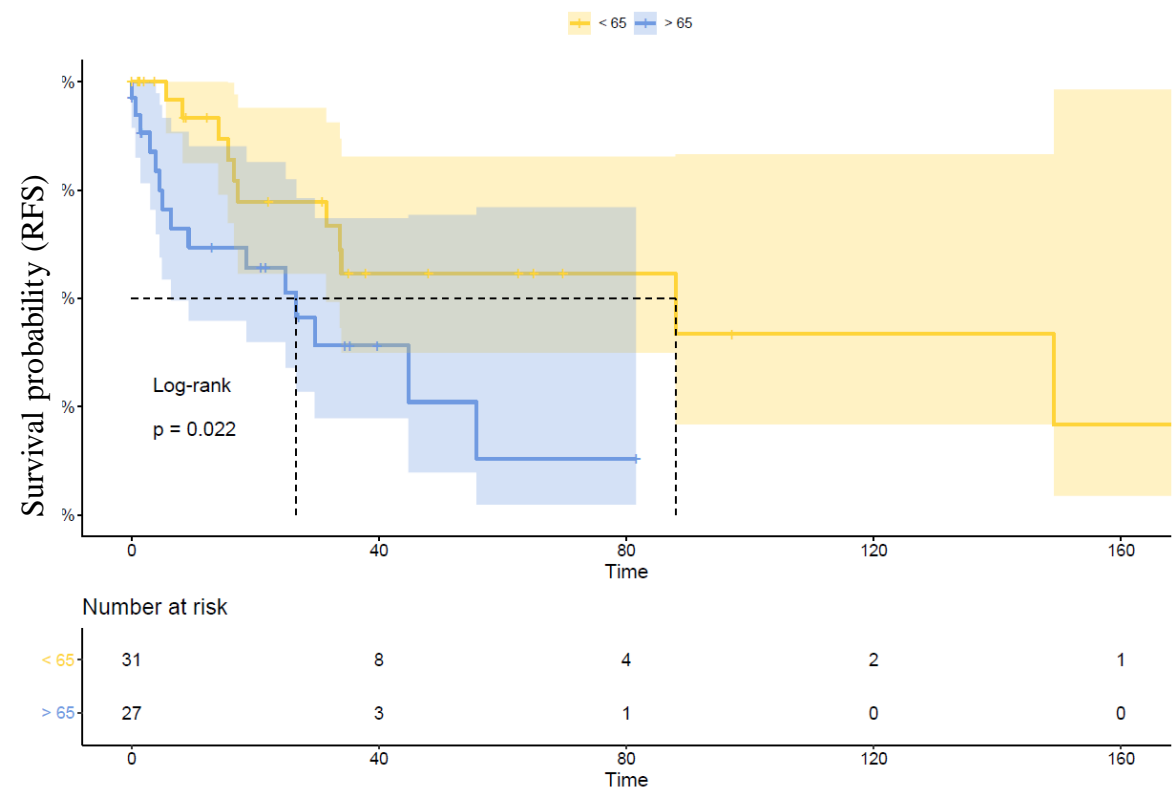

Lymph node status

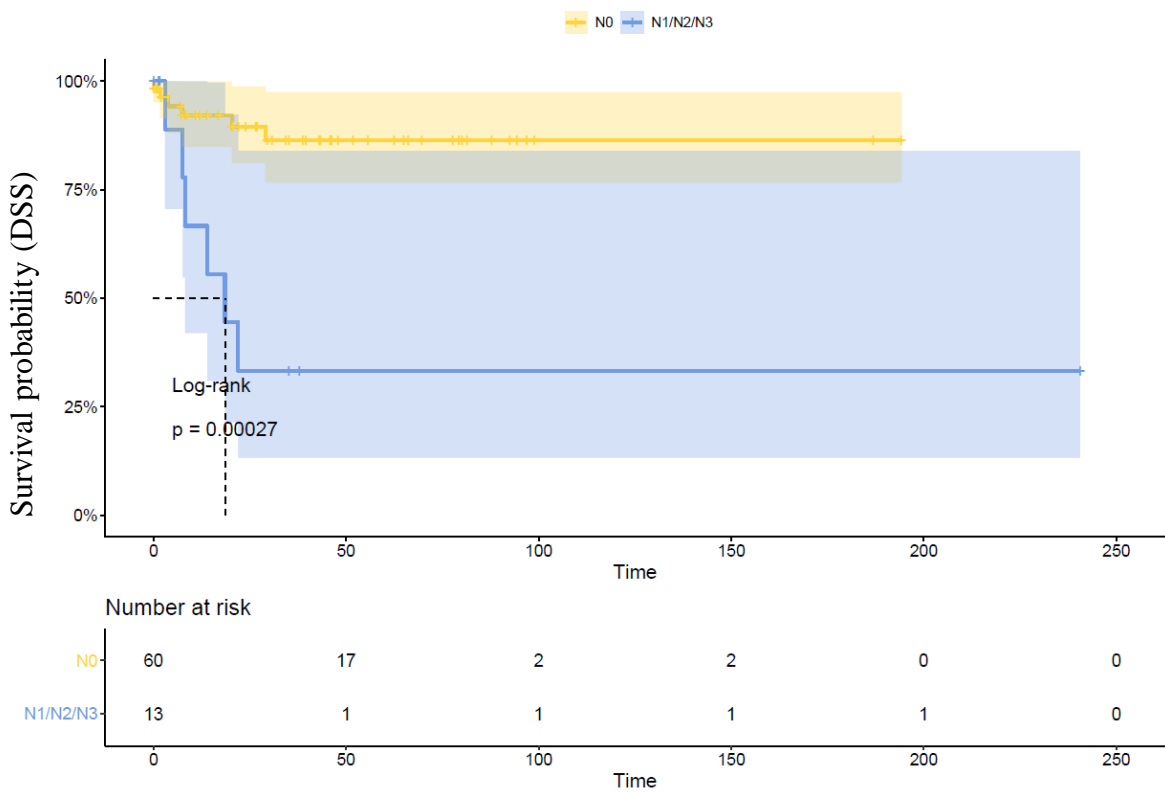

Supplement: Supplementary file 1 [file cancers-14-01683-s001.zip › Figure S4.pdf]

# Figure S5

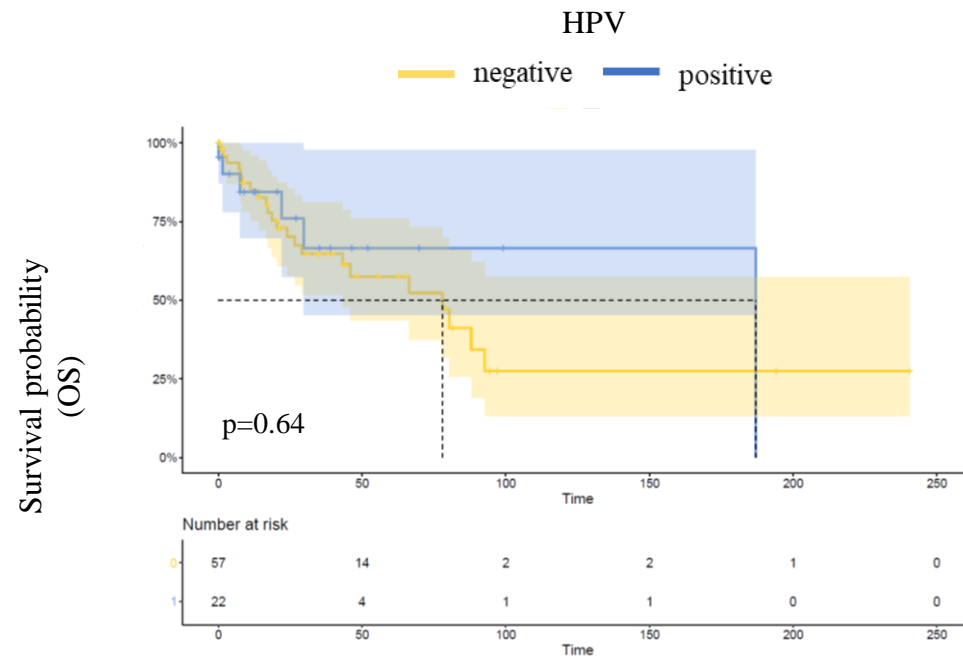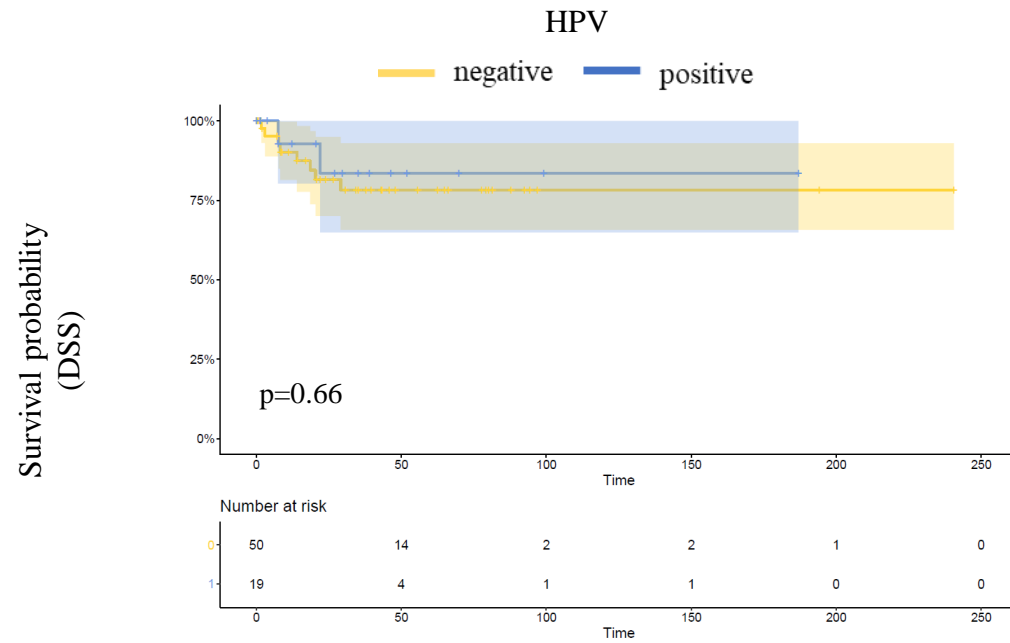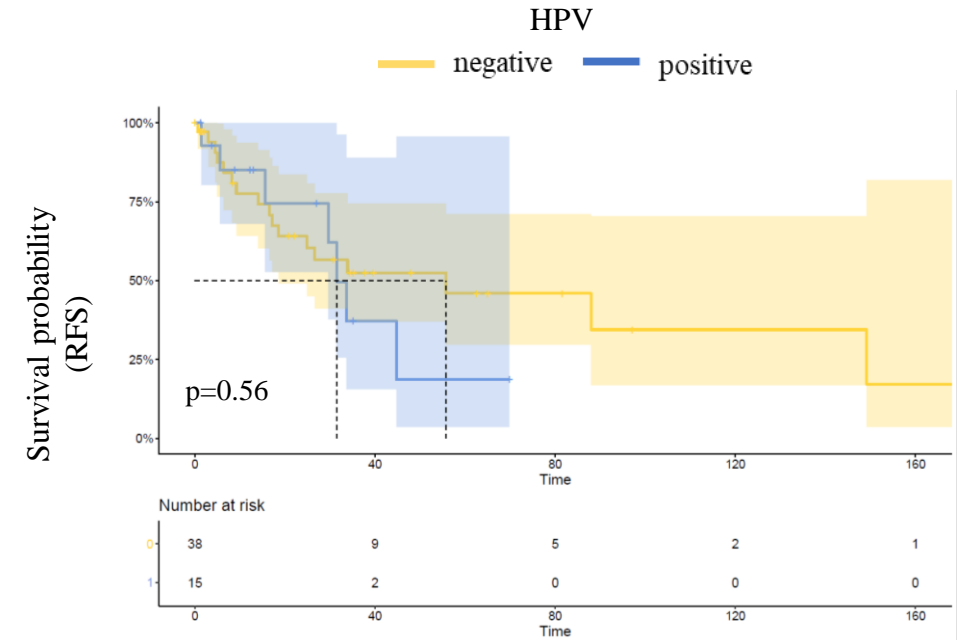

Supplement: Supplementary file 1 [file cancers-14-01683-s001.zip › Figure S5.pdf]
